# SOSTDC1 Nuclear Translocation Facilitates BTIC Maintenance and CHD1‐Mediated HR Repair to Promote Tumor Progression and Olaparib Resistance in TNBC

**DOI:** 10.1002/advs.202306860

**Published:** 2024-06-12

**Authors:** Qiaodan Deng, Jiankun Qiang, Cuicui Liu, Jiajun Ding, Juchuanli Tu, Xueyan He, Jie Xia, Xilei Peng, Siqin Li, Xian Chen, Wei Ma, Lu Zhang, Yi‐Zhou Jiang, Zhi‐Ming Shao, Ceshi Chen, Suling Liu, Jiahui Xu, Lixing Zhang

**Affiliations:** ^1^ Fudan University Shanghai Cancer Center & Institutes of Biomedical Sciences State Key Laboratory of Genetic Engineering Cancer Institutes Key Laboratory of Breast Cancer in Shanghai The Shanghai Key Laboratory of Medical Epigenetics Shanghai Key Laboratory of Radiation Oncology The International Co‐laboratory of Medical Epigenetics and Metabolism Ministry of Science and Technology Shanghai Medical College Fudan University Shanghai 200032 China; ^2^ Research Center for Translational Medicine Shanghai East Hospital Tongji University School of Medicine Shanghai 200120 China; ^3^ Department of Breast Surgery Shanghai Cancer Center and Cancer Institute Fudan University Shanghai 200032 P. R. China; ^4^ Department of Thyroid Breast and Vascular Surgery Xijing Hospital The Fourth Military Medical University Xi'an 710032 P. R. China; ^5^ Department of Breast Surgery Fudan University Shanghai Cancer Center Shanghai 200032 China; ^6^ Key Laboratory of Breast Cancer in Shanghai Department of Oncology Shanghai Medical College Fudan University Shanghai 200032 China; ^7^ Key Laboratory of Animal Models and Human Disease Mechanisms of Chinese Academy of Sciences and Yunnan Province Kunming Institute of Zoology Kunming 650201 China; ^8^ Academy of Biomedical Engineering & The Third Affiliated Hospital Kunming Medical University Kunming 650118 China; ^9^ Jiangsu Key Lab of Cancer Biomarkers Prevention and Treatment Collaborative Innovation Center for Cancer Medicine Nanjing Medical University Nanjing 211166 China

**Keywords:** homologous recombination, nuclear translocation, PARP inhibitor, SOSTDC1, triple‐negative breast cancer, tumor‐initiating cells

## Abstract

Breast tumor‐initiating cells (BTICs) of triple‐negative breast cancer (TNBC) tissues actively repair DNA and are resistant to treatments including chemotherapy, radiotherapy, and targeted therapy. Herein, it is found that a previously reported secreted protein, sclerostin domain containing 1 (SOSTDC1), is abundantly expressed in BTICs of TNBC cells and positively correlated with a poor patient prognosis. SOSTDC1 knockdown impairs homologous recombination (HR) repair, BTIC maintenance, and sensitized bulk cells and BTICs to Olaparib. Mechanistically, following Olaparib treatment, SOSTDC1 translocates to the nucleus in an importin‐α dependent manner. Nuclear SOSTDC1 interacts with the N‐terminus of the nucleoprotein, chromatin helicase DNA‐binding factor (CHD1), to promote HR repair and BTIC maintenance. Furthermore, nuclear SOSTDC1 bound to β‐transducin repeat‐containing protein (β‐TrCP) binding motifs of CHD1 is found, thereby blocking the β‐TrCP‐CHD1 interaction and inhibiting β‐TrCP‐mediated CHD1 ubiquitination and degradation. Collectively, these findings identify a novel nuclear SOSTDC1 pathway in regulating HR repair and BTIC maintenance, providing insight into the TNBC therapeutic strategies.

## Graphical abstract

In this study, SOSTDC1 is identified as a potential target mediating BTICs resistance to Olaparib. SOSTDC1 is highly expressed in TNBC tissues and especially in the BTIC population. Nuclear translocation of SOSTDC1 mediated by importin‐α stabilizes CHD1 protein from ubiquitination, thereby promoting HR repair, BTIC maintenance, and Olaparib resistance in TNBC.

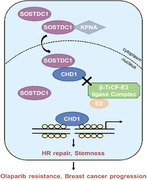

## 1 Introduction

Genomic instability is a characteristic of tumor cells.^[^ ^1^ ^]^ Tumor cells, especially tumor‐initiating cells (TICs) employ DNA repair mechanisms to facilitate cell survival under accumulating DNA damage and mutational load caused by replication stress, genotoxic chemicals, ionizing irradiation, and other endogenous and exogenous factors.^[^ ^2^ ^]^

Triple‐negative breast cancer (TNBC) is an aggressive subtype with limited targeted therapies,^[^ ^3^ ^]^ accounts for 12% to 17% of all breast cancers (BCs). Previous study has implied that DNA repair and Poly (ADP‐ribose) polymerase 1 (PARP1) protein expression may be upregulated in TNBC.^[^ ^4^, ^5^ ^]^ PARP inhibitors (PARPi) mainly inhibit PARP1‐mediated DNA single‐strand break repair, which can lead to synthetic lethality in tumors carrying germline mutations in either Breast cancer susceptibility gene 1/2 (BRCA1/BRCA2) or other homologous recombination (HR)  factors. PARPi also shows good efficacy whether used as monotherapy or in combination with other chemotherapy drugs in more common cancers that share this repair defect (homologous recombination deficiency, HRD).^[^ ^6^, ^7^, ^8^ ^]^ Olaparib is the first PARPi being approved for the treatment of TNBC patients with BRCA1/BRCA2 mutations.^[^ ^9^, ^10^, ^11^, ^12^, ^13^ ^]^ The function of PARP1 in HR repair and TNBC progression^[^ ^14^, ^15^, ^16^ ^]^ further supports the possibility of expanding the use of PARPi and there are several clinical trials or preclinical trials exploring the effects of PARPi in HR‐proficient TNBC.^[^ ^5^, ^17^ ^]^ However, Similar to other targeted therapies, PARPi resistance caused by many factors arises in advanced tumors.^[^ ^6^, ^7^, ^18^, ^19^, ^20^ ^]^ Therefore, the field is currently striving toward a better understanding of resistance to these agents and possible methods of overcoming this effect.

Tumor‐initiating cells (TICs) are reported responsible for tumor relapse and resistance to radio‐ and chemo‐therapy.^[^ ^21^ ^]^ Furthermore, in several tumor types, TICs exhibit increased DNA damage repair as well as resistance to PARPi.^[^ ^22^, ^23^, ^24^, ^25^, ^26^, ^27^ ^]^ Breast tumor‐initiating cells (BTICs) are highly heterogeneous and are found as three phenotypes; CD24^−^CD44^+^ BTICs are in a mesenchymal‐like state, ALDH^+^ BTICs are in an epithelial‐like state, as well as a small overlapping cell population (ALDH^+^CD24^−^CD44^+^) with the greatest tumorigenic capacity.^[^ ^28^, ^29^, ^30^ ^]^ Previous studies have showed that, in both BRCA1‐mutant and BRCA1‐wild‐type TNBCs, BRCA1 and BRCA2 expression did not significantly differ between BTICs and non‐BTICs. Dysfunction of BRCA1 resulted in the accumulation of ALDH1‐positive/ER‐negative stem/progenitor cells in mammary tissue,^[^ ^31^ ^]^ while down‐regulation of BRCA1 resulted in significant increase of the breast cancer stem cell ‐like populations.^[^ ^32^ ^]^ However, another HR regulator RAD51 upregulated in ALDH^+^ BTICs and mediated BTICs resistant to PARP inhibitors^[^ ^33^, ^34^ ^]^ suggesting that increased HR repair efficiency bypassed BRCA1 function might mediate BTICs resistant to PARPi. A greater understanding of these mechanisms would provide for the identification of more TNBC therapeutic targets, with the potential for increased effectiveness of PARPi.

Sclerostin domain containing protein 1 (SOSTDC1) is a secreted protein which regulates WNT and BMP signaling pathways.^[^ ^35^ ^]^ The expression of SOSTDC1 is downregulated in many primary tumors associated with the hypermethylation of promoter.^[^ ^36^, ^37^ ^]^ However, the function of tumor‐associated SOSTDC1 is controversial, with most studies demonstrating SOSTDC1 to inhibit proliferation and invasion of tumor cells^[^ ^38^, ^39^, ^40^, ^41^ ^]^ and another study demonstrating SOSTDC1 to promote invasion and liver metastasis.^[^ ^42^ ^]^ No reports have demonstrated SOSTDC1 to affect TICs and described the intracellular roles of SOSTDC1.

Here, we demonstrate that SOSTDC1 is highly expressed in TNBC cells, especially in CD24^−^CD44^+^ ALDH^+^ BTICs. SOSTDC1 expression is positively related to tumor malignancy and a poor patient prognosis in TNBC patients. In response to DNA damage, SOSTDC1 translocates to the nucleus in an importin‐α dependent manner and interacts with chromatin helicase DNA‐binding factor (CHD1), which is a nucleoprotein regulating open chromatin around the DSBs. SOSTDC1‐CHD1 interaction inhibits β‐TrCP‐mediated CHD1 ubiquitination and degradation, thereby promoting HR repair, BTIC maintenance, and Olaparib resistance. SOSTDC1 knockdown sensitizes TNBC cells to Olaparib in vivo. Collectively, these results imply that targeting SOSTDC1 may potentially be effective for treating TNBC.

## 2 Results

### 2.1 SOSTDC1 Is Highly Expressed in TNBC Tissues and Especially in The BTIC Population

**Figure 1:**
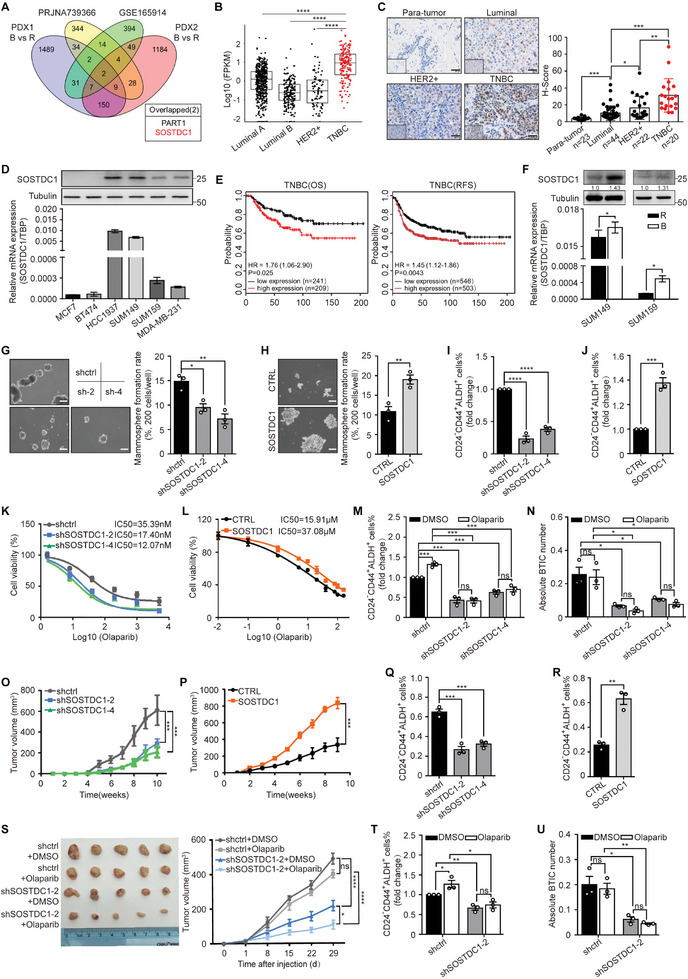
SOSTDC1 is highly expressed in TNBC and positively correlated with malignancy and Olaparib resistance. A) Overlap analysis of the upregulated genes in TNBC tumors (TNBC versus non‐TNBC >two‐fold, PRJNA739366), BTICs from two TNBC PDXs (BTICs B) versus the rest non‐BTICs (R), PRJNA376644) and an Olaparib‐resistant gene cluster of TNBC cells (GSE165914). (B) SOSTDC1 mRNA expression level of patients in the TCGA database with different molecular subtypes of breast cancer. Statistical significance was determined using Mann–Whitney U‐tests. C) Immunohistochemistry analysis of SOSTDC1 expression in para‐tumor tissues and tumor tissues from BC patients. Representative images of different molecular subtypes were shown and H‐Scores were calculated. Data represent the mean ± SEM, following unpaired Student's two‐sided *t*‐tests. ^*^ *p* < 0.05; ^**^ *p* < 0.01; ^***^ *p* < 0.001. Scale bar, 100 µm. D) mRNA and protein expression of SOSTDC1 in different subtypes of BC cell lines. E) Overall survival and relapse‐free survival of TNBC patients in Kaplan‐Meier Plotter with high or low SOSTDC1 expression. Statistical significance was assessed using a log‐rank test. F) mRNA and protein expression of SOSTDC1 in sorted BTICs (B) and the rest non‐BTICs (R) of SUM149 and SUM159. ^*^ *p* < 0.05 (unpaired Student's two‐sided *t*‐tests). G,H) Mammosphere formation assays of SOSTDC1‐knockdown SUM149 cells G) or SOSTDC1‐overexpressing SUM159 cells H). Representative images were shown and data were presented as mean ± SEM. Statistical significance was determined using the one‐way ANOVA or unpaired Student's two‐sided *t*‐tests. ^*^ *p* < 0.05; ^**^ *p* < 0.01. Scale bar, 100 µm. I–J) The percentage of BTIC population in SOSTDC1‐knockdown SUM149 cells I) or SOSTDC1‐overexpressing SUM159 cells J). Data were presented as mean ± SEM, following the one‐way ANOVA or unpaired Student's two‐sided *t*‐tests. ^***^ *p* < 0.001; ^****^ *p* < 0.0001. K,L) Survival analysis of SOSTDC1‐knockdown SUM149 cells K) or SOSTDC1‐overexpressing SUM159 cells L) in response to Olaparib; *n* =  3 independent biological samples. M,N) The percentage M) and absolute number N) of BTIC population in SOSTDC1‐knockdown SUM149 cells treated with 10 nmol L^−1^ Olaparib for 5d. Data were presented as mean ± SEM, following the two‐way ANOVA. ^*^ *p* < 0.05; ^***^ *p* < 0.001; ns, not significant. O) Tumor volumes of SOSTDC1‐knockdown SUM149 xenografts (four mice per group, two sites each mouse, 5000 cells per site). Data were presented as mean ± SEM. ^***^ *p* < 0.001 (the one‐way ANOVA). P) Tumor volumes of SOSTDC1‐overexpressing MDA‐MB‐231 xenografts (three mice per group, two sites each mouse, 1 million cells per site). Data were presented as mean ± SEM. ^***^ *p* < 0.001 (unpaired Student's two‐sided *t*‐tests.). Q,R) The percentage of BTIC population of SOSTDC1‐knockdown SUM149 xenografts Q) or SOSTDC1‐overexpressing MDA‐MB‐231 xenografts R). Data were presented as mean ± SEM, following the one‐way ANOVA or unpaired Student's two‐sided *t*‐tests. ^**^ *p* < 0.01; ^***^ *p* < 0.001. S–U) The effect of Olaparib treatment (50 mg kg^−1^, intraperitoneally, once a day) on SUM149 xenograft growth (SOSTDC1‐knockdown and control) in nude mice (three mice per group, two sites each mouse, 0.8 million cells per site). Tumor volumes and a representative image of tumors were shown in S). The percentage of the BTIC population in each group was shown in (T), and absolute BTIC numbers of each group are shown in (U). Data were presented as mean ± SEM, following the two‐way ANOVA. ^*^ *p* < 0.05; ^**^ *p* < 0.01; ^****^ *p* < 0.0001; ns, not significant.

Our previous study showed that, in both BRCA1‐mutant and BRCA1–wild‐type TNBCs, BTICs are relatively resistant to PARPi. To identify potential targets mediating BTICs resistance to PARPi and to explore the underlying regulatory mechanisms, we analyzed and integrated the transcriptional profiles of TNBC tumors, ALDH^+^CD24^−^CD44^+^ BTICs (BTICs thereafter) from two TNBC patient‐derived xenografts (PDXs),^[^ ^30^, ^43^ ^]^ and an Olaparib‐resistant gene cluster of TNBC cells.^[^ ^44^ ^]^ Two genes were upregulated in common (**Figure** **1**A). We focused on the protein‐coding gene SOSTDC1. Consistent with genetic profile analysis, SOSTDC1 was highly expressed in TNBC of The Cancer Genome Atlas (TCGA) samples (Figure 1B). In addition, bioinformatics analysis results had confirmed that SOSTDC1 expression was higher in BRCA1‐mutant breast tumor patients (Figure S1A, Supporting Information).

We then set out to detect the expression of SOSTDC1, and found that the expression of SOSTDC1 was higher in TNBC patient tumor samples (Figure 1C; Figure S1B, Supporting Information) as well as TNBC cell lines (Figure 1D). The relationships between SOSTDC1 level and clinical characteristics were evaluated. SOSTDC1 expression was inversely associated with tumor size (*p* = 0.037), grade (*p* = 0.019), ER status (*p* < 0.001), PR status (*p* = 0.001), and HER2 status (*p* = 0.007) (Table S1, Supporting Information), but positively associated with poor overall survival (OS) (*p* = 0.025) and poor relapse‐free survival (RFS) (*p* = 0.0043) of TNBC patient (Figure 1E). Similar effects were not observed in non‐TNBC patients (Figure S1C, Supporting Information). Cluster analysis identified 6 TNBC subtypes displaying unique gene expression and ontologies, including 2 basal‐like (BL1 and BL2), an immunomodulatory (IM), a mesenchymal (M), a mesenchymal stem–like (MSL), and a luminal androgen receptor (LAR) subtype.^[^ ^45^ ^]^ High SOSTDC1 expression was specifically related to poor OS in BL1 subtype patients with elevated DNA damage response pathways and proliferation pathways (Figure S1D, Supporting Information). Further, BTICs were sorted from the BRCA1‐mutant cell line, SUM149, and the BRCA1‐wildtype cell line, SUM159. SOSTDC1 was increased in the BTIC population of both cell lines compared with the non‐BTIC populations (Figure 1F), altogether implying an unusual pro‐tumorigenic role for SOSTDC1 in TNBC especially in BTICs.

### 2.2 SOSTDC1 Promotes TNBC Malignancy and Olaparib Resistance

SOSTDC1 function was assessed by silencing or overexpressing in TNBC cells. Stable‐SOSTDC1‐knockdown cell lines were established for SUM149 and HCC1937 (Figure S2A, Supporting Information). Stable‐SOSTDC1‐overexpression cell lines were established for SUM159 and MDA‐MB‐231 (Figure S2B, Supporting Information). SOSTDC1 expression was found to be positively related to TNBC cell proliferation (Figure S2C–F, Supporting Information), mammosphere formation (Figure 1G,H; Figure S2G,H, Supporting Information), BTIC population enrichment (Figure 1I,J, Figure S2I–L, Supporting Information), and Olaparib resistance (Figure 1K–L; Figure S2M, Supporting Information). Importantly, SOSTDC1‐knockdown rendered the BTIC population sensitive to Olaparib as judged by a decrease in absolute BTIC numbers (Figure 1M,N; Figure S2N, Supporting Information). Moreover, in vivo xenograft experiments showed that SOSTDC1 knockdown significantly inhibited tumor growth (Figure 1O; Figure S2O), meanwhile SOSTDC1 overexpression promoted tumor outgrowth (Figure 1P; Figure S2P, Supporting Information). BTIC population was decreased in SOSTDC1‐knockdown xenografts but increased in SOSTDC1‐overexpressing xenografts (Figure 1Q–R; Figure S2Q–R, Supporting Information).

To investigate whether BRCA1 status affected the function of SOSTDC1, we established SOSTDC1‐overexpressing SUM149 cells (BRCA1‐mutant) and SOSTDC1‐knockdown SUM159 cells (BRCA1‐wildtype, Figure S3A,B, Supporting Information). Consistent with our results above, SOSTDC1 overexpression made SUM149 cells more insensitive to Olaparib and SOSTDC1 knockdown sensitized SUM159 cells to Olaparib (Figure S3C, Supporting Information), regardless of BRCA1 status. These results indicated that cell sensitivity to Olaparib regulated by SOSTDC1 was not entirely dependent on BRCA1 status.

The therapeutic effect of Olaparib was also evaluated in SOSTDC1‐knockdown xenografts. We found that SOSTDC1‐knockdown and treatment with Olaparib significantly decreased tumor volume (Figure 1S), the BTIC population (Figure 1T; Figure S2S, Supporting Information), and absolute BTIC numbers (Figure 1U). These results demonstrated that SOSTDC1 mediated BTIC maintenance and Olaparib resistance in TNBC. Targeting SOSTDC1 might increase the therapeutic effect of PARPi.

### 2.3 Importin‐α‐Mediated SOSTDC1 Nuclear Translocation Promotes BTIC Maintenance and Olaparib Resistance

Since SOSTDC1 has been reported to function as a secreted protein, we treated the parental BC cells with culture supernatants of SOSTDC1‐overexpressing cells (Figure S4A, Supporting Information). However, the culture supernatants had no significant effect on cell proliferation (Figure S4B,C, Supporting Information), BTIC enrichment (Figure S4D, Supporting Information), or Olaparib resistance (Figure S4E, Supporting Information), indicating that the observed SOSTDC1 biological functions (those described above) are dependent on intracellular expression.

**Figure 2:**
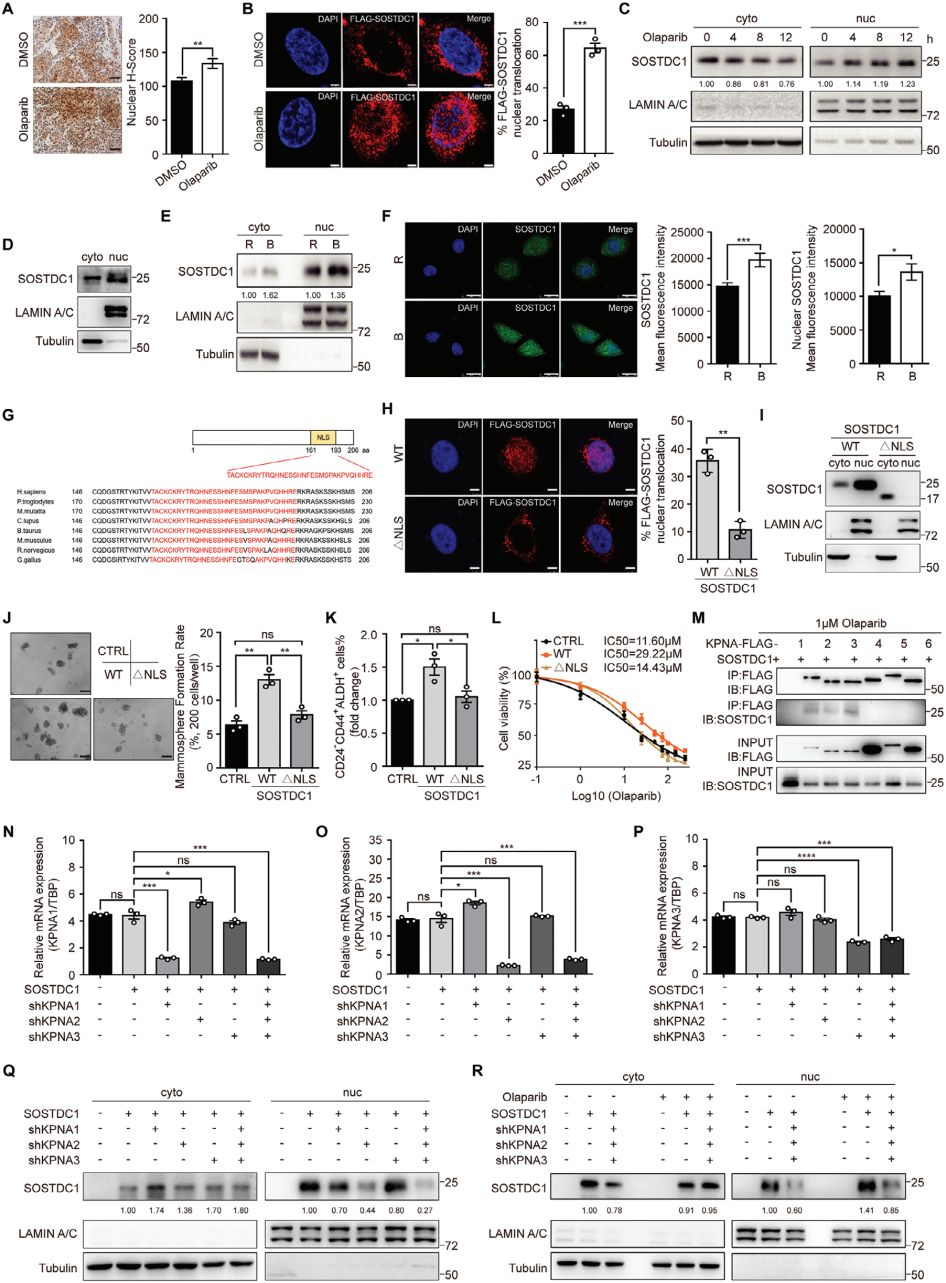
Importin‐α‐mediated SOSTDC1 nuclear translocation promotes BTIC maintenance and Olaparib resistance of TNBC cells. A) Immunohistochemistry analysis of nuclear SOSTDC1 expression in SUM149 xenografts treated with DMSO or Olaparib. Representative images were shown and H‐Scores were calculated. Data were presented as mean ± SEM. Significance was calculated using unpaired Student's two‐sided *t*‐tests. ^**^ *p* < 0.01. Scale bar, 100 µm. B) SOSTDC1‐overexpressing SUM159 cells were treated with DMSO or 5 µmol L^−1^ Olaparib for 12 h before immunofluorescence analysis using anti‐FLAG antibody. Representative images were shown and percentages of SOSTDC1 nuclear translocation were calculated. Data were presented as mean ± SEM, following unpaired Student's two‐sided *t*‐tests. ^***^ *p* < 0.001. Scale bar, 5 µm. C) Time‐dependent analysis of the expression of cytoplasmic and nuclear SOSTDC1 by western blotting after 1 µmol L^−1^ Olaparib treatment in SOSTDC1‐overexpressing SUM159 cells. D) Analysis of the endogenous expression of cytoplasmic and nuclear SOSTDC1 by western blotting in SUM149 cells. E) Analysis of the expression of cytoplasmic and nuclear SOSTDC1 by western blotting in sorted BTICs (B) and the rest non‐BTICs (R) of SOSTDC1‐overexpressing SUM159 cells. F) Immunofluorescence analysis in sorted BTICs (B) and the rest non‐BTICs (R) of SUM149 cells using anti‐SOSTDC1 antibody. Representative images were shown and the fluorescence intensities of SOSTDC1 and nuclear SOSTDC1 were measured. Data were presented as mean ± SEM. Significance was calculated using unpaired Student's two‐sided *t*‐tests. ^*^ *p* < 0.05; ^***^ *p* < 0.001. Scale bar, 20 µm. G) Schematic of the location and sequence of the predicted SOSTDC1 nuclear localization sequences. H,I) Wild‐type (WT) or NLS‐deleted mutant (ΔNLS) SOSTDC1‐overexpressing SUM159 cells were treated with DMSO or 5 µmol L^−1^ Olaparib for 12 h. Immunofluorescence staining using anti‐FLAG antibody H) analyzed the percentage of SOSTDC1 nuclear translocation (Data were presented as mean ± SEM, following unpaired Student's two‐sided *t*‐tests. ^**^ *p* < 0.01. Scale bar, 5 µm) and western blotting I) analyzed the expression of cytoplasmic and nuclear SOSTDC1. J) Mammosphere formation assays of wild‐type (WT) or NLS‐deleted mutant (ΔNLS) SOSTDC1‐overexpressing SUM159 cells. Representative images were shown and data were presented as mean ± SEM. Statistical significance was determined using the one‐way ANOVA. ^**^ *p* < 0.01; ns, not significant. Scale bar, 100 µm. K) The percentage of BTIC population in wild type (WT) or NLS‐deleted mutant (ΔNLS) SOSTDC1‐overexpressing SUM159 cells. Data were presented as mean ± SEM, following the one‐way ANOVA. ^*^ *p* < 0.05; ns, not significant. L) Survival analysis of wild type (WT) or NLS‐deleted mutant (ΔNLS) SOSTDC1‐overexpressing SUM159 cells in response to Olaparib; *n*  =  3 independent biological samples. M) Co‐IP assay detected the interaction between SOSTDC1 and protein members of the importin‐α. 293T cells were co‐transfected with SOSTDC1 and the indicated FLAG‐tagged KPNA vectors for 36 h, then exposed to 1µµ Olaparib for 4 h. The cell lysates were subjected to co‐IP experiments with anti‐FLAG magnetic beads; IB, immunoblot. N–P) mRNA expression of KPNA1 N), KPNA2 O) or KPNA3 P) in SOSTDC1‐overexpressing SUM159 cells with KPNA1 and KPNA2 and KPNA3 knockdown separately or simultaneously. Data were presented as mean ± SEM, following the one‐way ANOVA. ^**^ *p* < 0.01, ^***^ *p* < 0.001, ^****^ *p* < 0.0001. Q) Analysis of the expression of cytoplasmic and nuclear SOSTDC1 after 1 µmol L^−1^ Olaparib treatment for 12 h by western blotting in SOSTDC1‐overexpressing SUM159 cells with KPNA1, KPNA2, KPNA3 knockdown separately or simultaneously. R) Analysis of the expression of cytoplasmic and nuclear SOSTDC1 after DMSO or 1 µmol L^−1^ Olaparib treatment for 12 h by western blotting in SOSTDC1‐overexpressing SUM159 cells with KPNA1, KPNA2, and KPNA3 knockdown.

Intriguingly, SOSTDC1 could be detected in both cytoplasm and cell nucleus, and nuclear SOSTDC1 expression was significantly upregulated after Olaparib treatment in xenografts (**Figure** **2**A), which was further confirmed in TNBC cell lines (Figure 2B,C) as well as in U2OS cells (Figure S5A, Supporting Information). Secreted proteins have been shown to transport to the nucleus to regulate some important processes such as cell proliferation and differentiation, DNA replication, DNA repair, etc.^[^ ^46^ ^]^ However, there were few reports about the nuclear translocation of secreted proteins involved in the WNT and BMP signal pathway. We here confirmed the nuclear translocation of SOSTDC1 in parental TNBC cells (Figure 2D). Moreover, we observed a higher level of nuclear SOSTDC1 protein in BTICs (Figure 2E,F). These results suggested a biological association of nuclear SOSTDC1 with BTIC maintenance and Olaparib resistance.

Bioinformatic analysis identified a conserved nuclear localization signal (NLS) sequence within SOSTDC1 (Figure 2G). Deletion of the NLS not only significantly blocked the nuclear translocation of SOSTDC1 (Figure 2H,I), also reversed full‐length SOSTDC1‐induced mammosphere formation (Figure 2J), BTIC enrichment (Figure 2K; Figure S5B, Supporting Information) and Olaparib resistance (Figure 2L). It is widely accepted that importin‐α plays an indispensable role in protein nuclear import by recognition of the NLS.^[^ ^47^ ^]^ We found that SOSTDC1 may interact with protein members of the importin‐α family karyopherin (KPNA), including KPNA1, KPNA2, and KPNA3 (Figure S5C, Supporting Information). We verified the interactions upon Olaparib treatment (Figure 2M). Furthermore, we observed nuclear import of SOSTDC1 was modestly inhibited after knocking down KPNA proteins separately (Figure S5D,E, Supporting Information), but was clearly inhibited after knocking down these three importins simultaneously with or without Olaparib treatment (Figure 2N–R; Figure S5F, Supporting Information). Collectively, these results demonstrated importin‐α‐mediated SOSTDC1 nuclear translocation promotes BTIC maintenance and Olaparib resistance of TNBC cells.

### 2.4 Nuclear SOSTDC1 Promotes HR Repair

**Figure 3:**
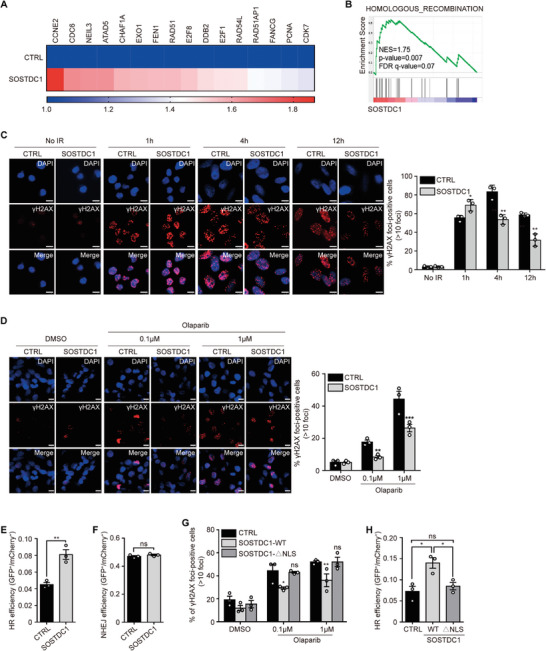
Nuclear SOSTDC1 promotes HR repair. A) Transcriptional profile analysis of the mRNA expression of genes associated with DNA repair in control and SOSTDC1‐overexpressing SUM159 cells. B) GSEA showed the enrichment for HR repair‐related genes with increased expression in SOSTDC1‐overexpressing SUM159 cells. C) Immunofluorescence analysis of γ‐H2AX foci at indicated times after irradiation (5 Gy) in SOSTDC1‐overexpressing SUM159 cells. Representative images were shown and percentages of γ‐H2AX foci‐positive cells (>10 foci) were quantified. ^*^ *p* < 0.05; ^**^ *p* < 0.01 (unpaired Student's two‐sided *t*‐tests). Scale bar, 10 µm. D) Immunofluorescence analysis of γ‐H2AX foci in SOSTDC1‐overexpressing SUM159 cells treated with DMSO or Olaparib (0.1 µmol L^−1^, 1 µmol L^−1^) for 12 h. Representative images were shown and percentages of γ‐H2AX foci‐positive cells (>10 foci) were quantified. ^**^ *p* < 0.01; ^***^ *p* < 0.001 (unpaired Student's two‐sided t‐tests). Scale bar, 10 µm. E) Analysis of HR efficiency in SOSTDC1‐overexpressing cells using the DR‐GFP reporter assay. ^**^ *p* < 0.01 (unpaired Student's two‐sided *t*‐tests). F) Analysis of NHEJ efficiency in SOSTDC1‐overexpressing cells using the pBigT‐neo‐GFP reporter assay. Data were presented as mean ± SEM. Significance was calculated using unpaired Student's two‐sided t‐tests. ns, not significant. G) Immunofluorescence analysis of γ‐H2AX foci in wild type (WT) or NLS‐deleted mutant (ΔNLS) SOSTDC1‐overexpressing SUM159 cells treated with DMSO or Olaparib (0.1 µmol L^−1^, 1 µmol L^−1^) for 12 h. Percentages of γ‐H2AX foci‐positive cells (>10 foci) were quantified. ^*^ *p* < 0.05; ^**^ *p* < 0.01; ns, not significant (the one‐way ANOVA). Scale bar, 10 µm. H) Analysis of HR efficiency in wild type (WT) or NLS‐deleted mutant (ΔNLS) SOSTDC1‐overexpressing cells using the DR‐GFP reporter assay. ^*^ *p* < 0.05; ns, not significant (the one‐way ANOVA).

To further explore the biological significance of SOSTDC1 nuclear translocation and its association with PARPi resistance, we analyzed the transcriptional profiles of SUM159‐CTRL and SUM159‐SOSTDC1 cells. A series of DNA repair‐related genes were upregulated in SOSTDC1 overexpressing cells (**Figure** **3**A). Gene set enrichment analysis (GSEA) and KEGG pathway analysis indicated that the HR repair pathway was significantly upregulated with SOSTDC1 overexpression (Figure 3B; Figure S6A, Supporting Information). However, we did not observe a consistent dysregulation of several classic HR regulator genes, including BRCA1, BRCA2, BARD1, and RAD51 (Figure S6B,C, Supporting Information). We examined γH2AX foci formation, which is a sensitive indicator of DSBs arising from uncompleted repair of base damage, in SOSTDC1‐overexpressing cells exposed to ionizing radiation (IR). In contrast to control cells, at 12 h after IR, SOSTDC1‐overexpressing cells exhibited shortened and diminished γH2AX foci formation (Figure 3C). Moreover, we observed that upon Olaparib treatment, SOSTDC1 overexpressing cells showed decreased γH2AX foci (Figure 3D), while SOSTDC1 knockdown resulted in an elevated level of γH2AX foci (Figure S6D,E, Supporting Information), suggesting that SOSTDC1 contributes to Olaparib induced DNA damage repair. In particular, we observed that SOSTDC1‐overexpressing cells showed better viability (Figure S6F,G, Supporting Information) and decreased γH2AX foci with Cisplatin treatment, indicating that SOSTDC1 may also function in the HR process in response to other DNA damaging drugs (Figure S6H, Supporting Information). Next, we utilized the integrated reporter assay for HR and non‐homologous end‐joining (NHEJ)^[^ ^48^ ^]^ to examine how SOSTDC1 promotes DNA repair. We observed significantly activated HR repair in SOSTDC1 overexpressing cells (Figure 3E; Figure S6I, Supporting Information), while only a minor increase in NHEJ efficiency (Figure 3F; Figure S6J, Supporting Information), declaring an important role of SOSTDC1 in the HR pathway. Importantly, deleting of the NLS recovered SOSTDC1 suppressed γH2AX foci formation upon Olaparib treatment (Figure 3G; Figure S6K, Supporting Information) and decreased the HR efficiency (Figure 3H; Figure S6L, Supporting Information). In addition, SOSTDC1 knockdown did not affect cell‐cycle distribution (Figure S7A, Supporting Information). Compared with PARP inhibitor or Cisplatin, SOSTDC1 knockdown did not impact cell sensibility to Docetaxel (DTX), which stops the growth of cancer cells by interfering with microtubules and blocking cell division (Figure S7B, Supporting Information). Collectively, these results suggested that nuclear SOSTDC1 promoted DNA repair, specifically HR repair, upon genotoxic stress.

### 2.5 Nuclear SOSTDC1 Interacts with CHD1 to Facilitate HR Repair, BTIC Maintenance, and Olaparib Resistance

**Figure 4:**
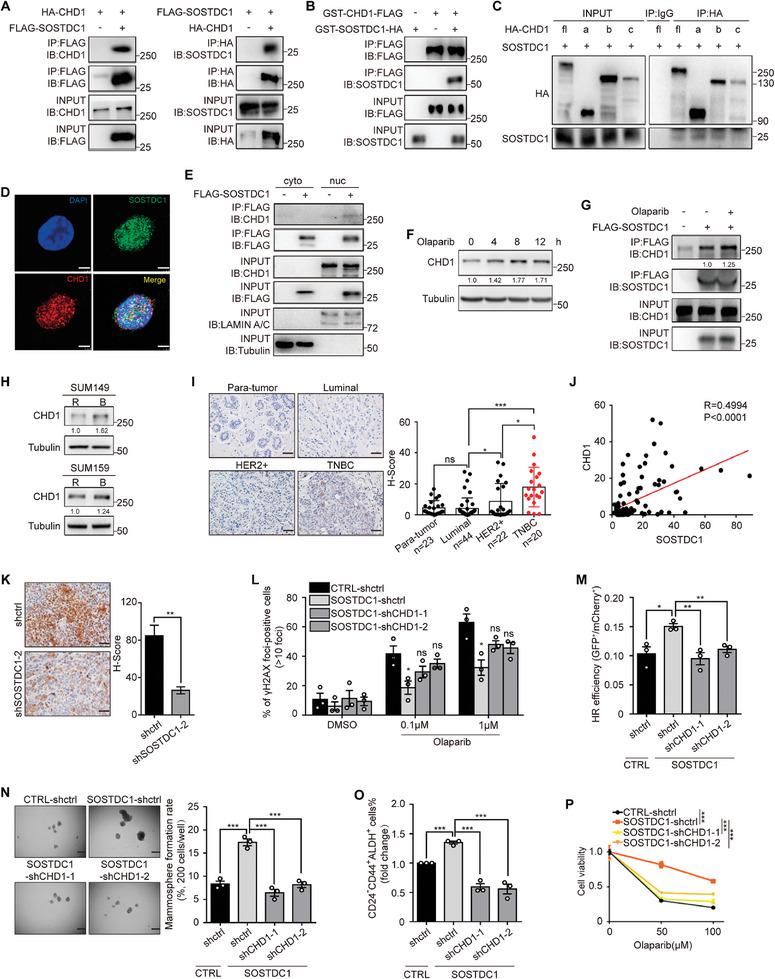
Nuclear SOSTDC1 interacts with CHD1 to promote HR repair, BTIC maintenance, and Olaparib resistance. A) Co‐IP assay detected the interaction between SOSTDC1 and CHD1. Vectors encoding HA‐tagged CHD1 or FLAG‐tagged SOSTDC1 were transfected into 293T cells, which were subjected to co‐IP experiments with magnetic beads coated with antibodies to FLAG or HA, followed by western blotting with the indicated antibodies. B) In vitro co‐IP assay detected the interaction between SOSTDC1 and CHD1. Purified HA‐tagged SOSTDC1 or FLAG‐tagged CHD1 recombinant proteins were subjected to co‐IP experiments with anti‐FLAG magnetic beads, followed by western blotting with the indicated antibodies. C) Co‐IP assay detected the interaction between SOSTDC1 and full length CHD1 (fl) or its three truncated mutants (a, b, c). D) Immunofluorescence analysis of the distribution of endogenous SOSTDC1 and CHD1 in SUM149 cells upon 1 µmol L^−1^ Olaparib treatment for 12 h. Scale bar, 5 µm. E) Co‐IP assay detected the interaction between nuclear SOSTDC1 and CHD1. Cytoplasmic and nuclear lysates from FLAG‐tagged SOSTDC1‐overexpressing SUM159 cells were prepared for co‐IP experiments with anti‐FLAG magnetic beads, followed by western blotting with the indicated antibodies. F) Time‐dependent analysis of the expression of CHD1 by western blotting after 1 µmol L^−1^ Olaparib treatment in SUM159 cells. G) Co‐IP assay detected the interaction between SOSTDC1 and CHD1 upon 1 µmol L^−1^ Olaparib treatment for 12 h. H) CHD1 protein expression in sorted BTICs (B) and the rest non‐BTICs (R) of SUM149 and SUM159. I) Immunohistochemistry analysis of CHD1 expression in para‐tumor tissues and tumor tissues from BC patients. Representative images of different molecular subtypes were shown and H‐Scores were calculated. Data were presented as mean ± SEM. Significance was calculated using unpaired Student's two‐sided *t*‐tests. ^*^ *p* < 0.05; ^***^ *p* < 0.001; ns, not significant. Scale bar, 100 µm. J) Analysis of the correlation between SOSTDC1 protein levels (related to Figure 1C) and CHD1 protein levels (related to Figure 4I) in BC patient tumor tissues. Statistical significance was determined by Pearson's correlation test. K) Immunohistochemistry analysis of CHD1 expression in SOSTDC1‐knockdown SUM149 xenografts. Representative images were shown and H‐Scores were calculated. Data were presented as mean ± SEM, following unpaired Student's two‐sided *t*‐tests. ^**^ *p* < 0.01. Scale bar, 100 µm. L) SOSTDC1‐overexpressing SUM159 cells with CHD1‐knockdown were treated with DMSO or Olaparib (0.1 µmol L^−1^, 1 µmol L^−1^) for 12 h, following immunofluorescence analysis of γ‐H2AX foci. Percentages of γ‐H2AX foci‐positive cells (>10 foci) were quantified. Data were presented as mean ± SEM, following the one‐way ANOVA. ^*^ *p* < 0.05; ns, not significant. M) Analysis of HR efficiency in SOSTDC1‐overexpressing cells with CHD1‐knockdown using the DR‐GFP reporter assay. Data were presented as mean ± SEM. Significance was calculated using the one‐way ANOVA. ^*^ *p* < 0.05, ^**^ *p* < 0.01. N) Mammosphere formation assays of SOSTDC1‐overexpressing SUM159 cells with CHD1‐knockdown. Data were presented as mean ± SEM, following the one‐way ANOVA. ^***^ *p* < 0.001. Scale bar, 100 µm. O) The percentage of BTIC population in SOSTDC1‐overexpressing SUM159 cells with CHD1‐knockdown. Data were presented as mean ± SEM, following the one‐way ANOVA. ^***^ *p* < 0.001. P) Cell viability determined by MTT assay in SOSTDC1‐overexpressing SUM159 cells with CHD1‐knockdown. *n*  =  3 independent biological samples.

To explore how nuclear SOSTDC1 participates in HR, we utilized the co‐IP/MS assay to screen for SOSTDC1‐interacting proteins, and identified a potential SOSTDC1‐interacted nucleoprotein, CHD1 (Figure S8A,B, Supporting Information). CHD1 chromatin helicase DNA‐binding factor (CHD1) regulates open chromatin around the DSB to facilitate the recruitment of HR proteins. CHD1 protects genome integrity at promoters to sustain hypertranscription and pluripotency of embryonic stem cells.^[^ ^49^, ^50^ ^]^ Loss of CHD1 elicits cellular sensitivity to PARP inhibition.^[^ ^51^, ^52^, ^53^ ^]^ The direct interaction between SOSTDC1 and CHD1 was confirmed by co‐IP assay (**Figure** **4**A) and especially by in vitro co‐IP assay (Figure 4B). CHD1 consists of three main signature domains: Double chromodomain located in the N‐terminal region, ATPase motor centered in the middle of the protein, and DNA‐binding domain located in the C‐terminal region.^[^ ^54^ ^]^ Three CHD1 truncated mutants (a, b, c) were constructed to verify the SOSTDC1‐binding domain (Figure S8C, Supporting Information). Only truncations containing the N‐terminus of CHD1 (Fragment a and b) could interact with SOSTDC1 (Figure 4C). IF staining showed a nuclear co‐localization of SOSTDC1 with CHD1 upon Olaparib treatment (Figure 4D). Nuclear rather than cytoplasmic SOSTDC1 interacted with endogenous CHD1 (Figure 4E). Upon Olaparib treatment, CHD1 protein level was upregulated (Figure 4F) and the SOSTDC1‐CHD1 interaction dramatically enhanced (Figure 4G). Taken together, these results manifested the nuclear SOSTDC1‐CHD1 interaction.

Although CHD1 mRNA level in TNBC was lower than in other subtypes (Figure S8D, Supporting Information), the CHD1 protein level was significantly upregulated in TNBC cell lines (Figure S8E, Supporting Information) especially in BTICs (Figure 4H), and in TNBC patient samples (Figure 4I). There was a strong correlation between SOSTDC1 and CHD1 in BC patient samples (Figure 4J). Besides, CHD1 protein was markedly downregulated in SOSTDC1‐knockdown xenografts (Figure 4K). Survival analysis showed that high CHD1 expression associated with poor OS (*p* = 0.089) in BC patients (Figure S8F, Supporting Information). These results revealed that CHD1 protein level is positively correlated with SOSTDC1 expression and TNBC malignancy.

To investigate whether SOSTDC1‐CHD1 interaction is important for SOSTDC1 function, we knocked down CHD1 in SOSTDC1‐overexpressing SUM159 cells (Figure S8G, Supporting Information). CHD1 knockdown reversed the effect of SOSTDC1 on cell proliferation (Figure S8H, Supporting Information), DNA repair (Figure 4L; Figure S8I, Supporting Information), and in particular HR repair (Figure 4M; Figure S8J, Supporting Information), mammosphere formation (Figure 4N), BTIC enrichment (Figure 4O; Figure S8K, Supporting Information), and Olaparib resistance (Figure 4P). These results suggested that CHD1 is the main downstream pathway of SOSTDC1 function.

### 2.6 Nuclear SOSTDC1 Stabilizes CHD1 by Blocking Ubiquitin‐Dependent Degradation

**Figure 5:**
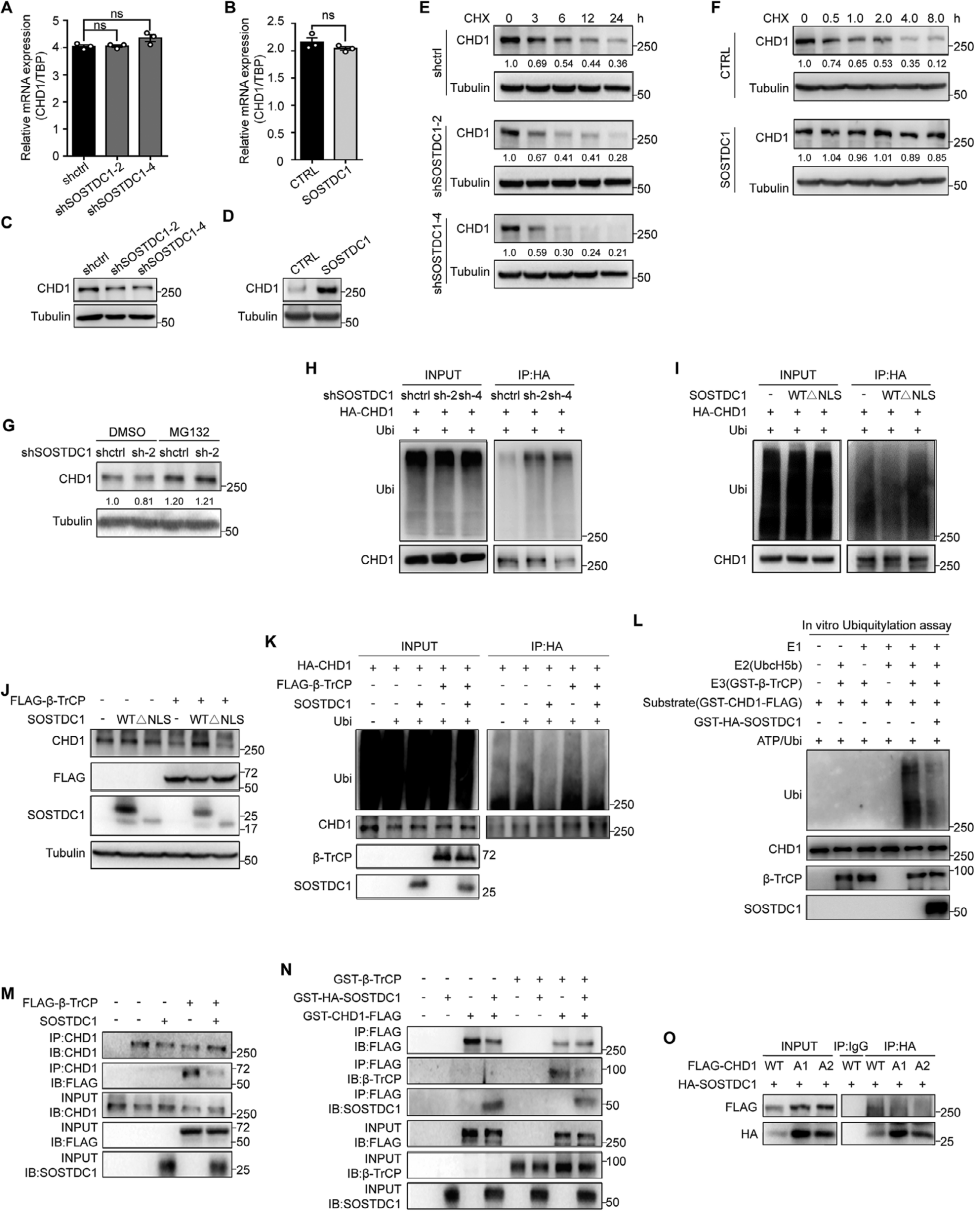
Nuclear SOSTDC1 inhibits β‐TrCP‐mediated ubiquitination and degradation of CHD1. A,B) CHD1 mRNA expression in SOSTDC1‐knockdown SUM149 cells (A) or SOSTDC1‐overexpressing SUM159 cells (B). C,D) CHD1 protein expression in SOSTDC1‐knockdown SUM149 cells C) or SOSTDC1‐overexpressing SUM159 cells D). E,F) Analysis of CHD1 protein level in SOSTDC1‐knockdown SUM149 cells E) or SOSTDC1‐overexpressing SUM159 cells F) treated with 100 µg mL^−1^ CHX for the indicated time. G) Analysis of CHD1 protein level in SOSTDC1‐knockdown SUM149 cells treated with 10 µmol L^−1^ MG132 for 4 h. H) 293T cells with HA‐tagged CHD1 overexpressing were transfected with SOSTDC1‐knockdown and ubiquitin‐encoding vectors. Cells were treated with 10 µmol L^−1^ MG132 for 8 h and collected for HA‐immunoprecipitation and analysis of CHD1 ubiquitination by western blotting. I) 293T cells with HA‐tagged CHD1 overexpressing were transfected with ubiquitin‐encoding, wild‐type (WT) or NLS‐deleted mutant (ΔNLS) SOSTDC1‐overexpressing vectors. Cells were treated with 10 µmol L^−1^ MG132 for 8 h and collected for HA‐immunoprecipitation and analysis of CHD1 ubiquitination by western blotting. J) Analysis of CHD1 protein level in Flag‐tagged β‐TrCP‐overexpressing cells with wild type (WT) or NLS‐deleted mutant (ΔNLS) SOSTDC1‐overexpressing. K) 293T cells with HA‐tagged CHD1 overexpressing were transfected with ubiquitin‐encoding, SOSTDC1‐encoding or FLAG‐tagged β‐TrCP‐overexpressing vectors. Cells were treated with 10 µmol L^−1^ MG132 for 8 h and collected for HA‐immunoprecipitation and analysis of CHD1 ubiquitination by western blotting. L) In vitro ubiquitination assay of β‐TrCP mediated CHD1 ubiquitination in the presence or absence of SOSTDC1. M) Co‐IP assay of the interaction between β‐TrCP and CHD1 in the presence or absence of SOSTDC1. N) In vitro co‐IP assay of the interaction between CHD1 and β‐TrCP in the presence or absence of SOSTDC1. O) Co‐IP assay of the interaction between SOSTDC1 and CHD1 mutants. Vectors encoding HA‐tagged SOSTDC1, FLAG‐tagged wild type, or the two β‐TrCP‐binding‐motif mutants of CHD1 were transfected into 293T cells, which were subjected to co‐IP experiments with anti‐HA magnetic beads, followed by western blotting with the indicated antibody.

We noticed that SOSTDC1 overexpression promoted while SOSTDC1 knockdown reduced CHD1 protein abundance, with little influence on CHD1 mRNA level (**Figure** **5**A–D), suggesting post‐translation effects of SOSTDC1 on CHD1. Consistent with our speculation, we found the degradation of endogenous CHD1 was accelerated by SOSTDC1 knockdown (Figure 5E) and decelerated by SOSTDC1 overexpression (Figure 5F). Further, we found that the inhibition of CHD1 induced by SOSTDC1 knockdown could be reversed by the proteasome inhibitor MG132 (Figure 5G), indicating the ubiquitin‐proteasome proteolysis regulation of CHD1, which is compatible with previous report.^[^ ^55^ ^]^ Moreover, the ubiquitylated CHD1 was significantly increased by SOSTDC1 knockdown (Figure 5H) and decreased by SOSTDC1 overexpression, which was counted on SOSTDC1 nuclear localization (Figure 5I). These results implied that nuclear SOSTDC1 stabilized CHD1 by blocking ubiquitin‐dependent degradation.

CHD1 has been reported to induce trimethyl lysine‐4 histone H3 (H3K4me3) DNA modifications, which activate transcription of the pro‐tumorigenic TNF–NF‐κB gene network.^[^ ^55^ ^]^ We found that SOSTDC1 positively regulated CHD1 downstream gene expression (Figure S9A,B, Supporting Information). Taken together, these results demonstrated that SOSTDC1 maintained CHD1 stabilization and promoted CHD1‐regulating genes expression.

### 2.7 Nuclear SOSTDC1 Inhibits β‐TrCP‐Mediated CHD1 Ubiquitination by Blocking the Interaction of β‐TrCP with CHD1

β‐TrCP is the substrate‐recognition subunit of the SCF^β‐TrCP^ (Skp1–Cullin1–F‐box protein) E3 ubiquitin ligase. The ligase is responsible for the ubiquitination and degradation of phosphorylated CHD1.^[^ ^55^ ^]^ Our Co‐IP results showed that β‐TrCP is also bound to the N‐terminus of CHD1, which contains two reported β‐TrCP consensus‐binding motifs (Figure S10A, Supporting Information). We then sought to clarify the role of SOSTDC1 in β‐TrCP‐mediated CHD1 degradation. Nuclear SOSTDC1 reversed the inhibition of CHD1 induced by β‐TrCP overexpression (Figure 5J). SOSTDC1 overexpression decreased β‐TrCP‐mediated CHD1 ubiquitination (Figure 5K), which was further confirmed by in vitro ubiquitylation assay (Figure 5L). SOSTDC1 had relatively little effect on β‐TrCP mRNA and protein expression (Figure S10B–E, Supporting Information) while significantly suppressed the interaction between CHD1 and β‐TrCP (Figure 5M,N). Two β‐TrCP consensus‐binding motifs – residues 23–28 (motif 1, DSGSAS) and 53–58 (motif 2, DSGSES), were involved in regulating β‐TrCP‐CHD1 interaction and CHD1 degradation.^[^ ^55^ ^]^ Co‐IP results demonstrated SOSTDC1 interacted with the β‐TrCP binding motifs of CHD1 (Figure 5O; Figure S10F, Supporting Information). Taken together, these results indicated that SOSTDC1 inhibited β‐TrCP‐mediated ubiquitination of CHD1 by blocking the interaction of CHD1 with β‐TrCP.

## 3 Discussion

PARPi has been approved for use in TNBC with disruptive mutations in BRCA1/BRCA2 or other HR factors. The roles of PARP1 in HR repair and the progression of TNBC suggest that PARPi can be further applied to HR‐proficient TNBC.^[^ ^6^, ^7^, ^8^ ^]^ However, like other drug therapies, resistance is still a significant issue. Previous reports have demonstrated that the sensitivity of breast cancer cells to PARPi could be influenced by a combination of factors such as HR deficiency (including BRCA1 status), drug efflux efficiency, activation of 53BP1 pathway, PARP activity,^[^ ^56^ ^]^ which indicated that BRCA1 status may not be the only indicator for PARPi sensitivity in breast cancer. Moreover, increased HR repair efficiency has been reported to mediate BTICs resistant to PARPi. In this study, we identified SOSTDC1 as a potential target mediating BTICs resistance to PARPi. SOSTDC1 translocates to the nucleus in an importin‐α dependent manner and interacts with CHD1 to promote its stability, thereby promoting HR repair, BTIC maintenance, and Olaparib resistance in TNBC (Figure S10G, Supporting Information). Targeting the SOSTDC1‐CHD1 axis may help us to improve the therapeutic effect of PARPi and further broaden the application of PARPi in TNBC.

SOSTDC1 is a secreted protein downregulated in many primary tumors and usually inhibits the tumor progression. However, in this study, we revealed a distinct expression pattern and function of SOSTDC1 in TNBC. First, SOSTDC1 was highly expressed in TNBC cells, high level of SOSTDC1 expression was associated with poor OS and poor RFS in TNBC patients. We found both mRNA expression and protein expression of SOSTDC1 in BRCA1‐wildtype breast cancer cells were lower than that in BRCA1‐mutant ones, which was also confirmed by public dataset analysis. Upregulated RAD51 in BRCA1‐mutant TNBC cells was reported to compensate for BRCA1 deficiency.^[^ ^57^ ^]^ We speculated higher SOSTDC1 expression may play similar roles. However, we could not draw definitive conclusions of the relationship between SOSTDC1 protein level and BRCA1 mutation since there was no available information on BRCA1 status in the TNBC patient tumor samples we used in this study. However, a high level of SOSTDC1 expression was associated with poor OS in the BL1 subtype of TNBC, which was featured with elevated DNA damage response (ATR/BRCA) pathways.^[^ ^45^ ^]^ SOSTDC1 overexpression promoted the transcriptional expression of a series of DNA repair‐related genes including RAD51. However, the classic HR regulators BRCA1, BRCA2, and BARD1 were not consistently dysregulated in SOSTDC1‐overexpressing or SOSTDC1‐knocking down cells. What's more, our results showed that SOSTDC1 regulated PARPi sensitivity in both BRCA1‐mutant and BRCA1‐wildtype TNBC cells, together implying SOSTDC1 function as an alternative HR‐related factor in TNBC.

Secreted proteins (e.g., FGFs, CCN proteins, IFNγ, EGF, and their receptors) are transported to the nucleus and involved in the regulation of important cellular processes such as cell proliferation, differentiation, DNA replication, and DNA repair.^[^ ^46^ ^]^ In this study, we demonstrated that SOSTDC1 could translocate into the nucleus in TNBC cells via interacting with KPNA1, KPNA2, and KPNA3. Secreted SOSTDC1 had no significant effect on TNBC cell proliferation and BTIC population. Olaparib treatment enhanced SOSTDC1 nuclear translocation. Further, BTICs expressed more nuclear SOSTDC1 than non‐BTICs. SOSTDC1 truncated mutant (ΔNLS) failed to enter the nucleus and lost the ability to promote the mammosphere formation, BTIC enrichment, HR repair, and Olaparib resistance. These results suggested that the nuclear SOSTDC1 is an independent indicator for BTIC maintenance and Olaparib resistance. However, it is important to note that whether there is any DNA damage that induced SOSTDC1 protein modification or whether there is other mechanism‐mediated SOSTDC1 nuclear translocation is not fully understood and requires further clarification.

Chromatin remodeling proteins play central roles in controlling the accessibility of DNA to enzymes and proteins involved in regulating gene transcription, DNA replication, and repair. CHD1 belongs to the family of chromodomain helicase DNA‐binding remodelers and was reported capable of assembling nucleosomes, remodeling chromatin structure, modulating histone turnover, and binding to H3K4me3 to activate gene transcription.^[^ ^54^ ^]^ CHD1 loss sensitizes cells to Olaparib and carboplatin treatment in vitro and in vivo.^[^ ^49^, ^53^ ^]^ However, CHD1 was not reported to be directly involved in regulating the expression of HR‐related genes. Either the genes concomitantly regulated by CHD1/H3K4me3 or the genes whose promoters are bound by CHD1 are not enriched in DNA repair, especially the HR repair pathway.^[^ ^55^ ^]^ At DSB, the regulation of chromatin structure has been described in the “access–repair–restore” model.^[^ ^58^ ^]^ CHD1 function during the “access” step to facilitates the generation of ssDNA and assists recruitment of CtIP to the damage site in the end resection process during HR‐mediated DSB repair. In our study, we supposed that SOSTDC1 mediated CHD1 protein stabilization enables cells to exhibit an HR‐repair‐enhanced phenotype, in which several DNA repair‐related genes were upregulated. SOSTDC1 and CHD1 were demonstrated to promote gene expression in the TNF‐NF‐κB pathway, and BRCA1 protein stabilization was regulated by many factors like HSP90,^[^ ^59^ ^]^ Cathepsin S,^[^ ^60^ ^]^ and NF‐κB signaling,^[^ ^61^, ^62^ ^]^ etc. However, the impact of SOSTDC1 and CHD1 expression on BRCA1 protein stabilization remained to be explored. Our results showed that SOSTDC1 overexpression stabilized CHD1 from β‐TrCP mediated CHD1 protein degradation^[^ ^55^ ^]^ and then activated CHD1‐regulating gene transcription. Mechanically, nuclear SOSTDC1 interacted with the β‐TrCP binding motifs of CHD1, thereby blocking the β‐TrCP–CHD1 interaction and inhibiting β‐TrCP‐mediated CHD1 ubiquitination and degradation. As the synthetic‐lethal interaction of PTEN and CHD1 is present in breast cancer,^[^ ^55^ ^]^ whether the regulation of SOSTDC1 on CHD1 affected by PTEN status? We observed SOSTDC1 overexpression promoted the stability of CHD1 protein in PTEN‐intact SUM159 cells, indicating that SOSTDC1 was an independent regulatory factor of CHD1 protein level. The relationship between SOSTDC1 expression and PTEN signaling remains under further investigation.

We mainly explored the expression patterns and functional mechanisms of SOSTDC1 on tumor cells. In fact, in the skeletal system, SOSTDC1 is essential for bone metabolism, bone density maintenance, and fracture healing.^[^ ^35^ ^]^ In the immune system, SOSTDC1 is secreted by a subpopulation of follicular helper *T*‐cells and is required for regulatory follicular *T*‐cell differentiation.^[^ ^63^ ^]^ Further functional analyses will be meaningful to explore the roles of SOSTDC1 in TNBC tumor microenvironment and bone metastasis.

## 4 Conclusion

In summary, this study suggests that SOSTDC1 promotes TNBC progression, as well as mediates Olaparib resistance of both BTICs and bulk‐tumor cells. Further, in response to DNA damage we identified SOSTDC1 translocates to the nucleus in an importin‐α dependent manner to interact with CHD1. SOSTDC1‐CHD1 interaction inhibited β‐TrCP‐mediated CHD1 ubiquitination and promoted HR repair, BTIC maintenance, and Olaparib resistance of TNBC cells. Therefore, by targeting SOSTDC1 or interfering SOSTDC1‐CHD1 interaction, it may be possible to increase the Olaparib sensitivity of TNBC cells. Unfortunately, currently there are no available inhibitors of SOSTDC1 or CHD1 and no available peptides for blockage of SOSTDC1‐CHD1 interaction. However, this study does provide promising targets by which to increase the therapeutic efficacy of PARPi for TNBC patient.

## 5 Experimental Section

### Cell lines and Cell Culture

Human breast cancer cell lines SUM149 and SUM159 were obtained from Asterland Bioscience, MCF7, T47D, BT474, HCC1954, HCC1937, and MDA‐MB‐231 were purchased from the American Type Culture Collection. SUM149 and SUM159 were maintained in Ham's F‐12 (Gibco) supplemented with 5% fetal bovine serum (FBS) (Gibco), 5 mg mL^−1^ insulin (Biosharp Life Science), 1 mg mL^−1^ hydrocortisone (Sigma–Aldrich), and 1% penicillin‐streptomycin (pen‐strep) (Beyotime Biotechnology). MCF7 was maintained in Eagle's minimum essential medium (Gibco) supplemented with 10% FBS, 10 mg mL^−1^ insulin, and 1% pen‐strep. T47D was maintained in RPMI 1640 (Gibco) with 10% FBS, 5 mg mL^−1^ insulin, and 1% pen‐strep. BT474, HCC1954, HCC1937 were maintained in RPMI 1640 with 5% FBS and 1% pen‐strep. 293T, MDA‐MB‐231 were maintained in DMEM (Gibco) with 10% FBS and 1% pen‐strep. All the cell lines were authenticated by STR profiling, confirmed to be mycoplasma‐free, and cultured in incubators (37 °C, 5% CO_2_).

### Tumorigenicity in Nude Mice

Breast cancer cells were injected with Matrigel into the fourth mammary fat pads of four‐week‐old female nude mice purchased from Vitalriver. The mice were housed in standard animal cages under a Specific‐pathogen‐free facility at 23–25 °C on a 12 h light/ dark cycle in the Department of Laboratory Animal Science of Fudan University. The in vivo treatment of Olaparib started when the average diameter of xenograft tumors reached to 3 mm. Mice in each group were further divided into another two groups and treated with DMSO or Olaparib (50 mg kg^−1^, once a day) for 29 days intraperitoneally. The tumor sizes were measured with a caliper and calculated as length × width × width/2 and monitored weekly. Mice were sacrificed when the diameter of tumors reached 10–15 mm.

### Plasmid Construction and Lentivirus Transfection

SOSTDC1 full‐length cDNA and its NLS‐deleted mutant with FLAG tag were cloned into the lentiviral vector pSIN (Addgene). FLAG‐tagged β‐TrCP, SOSTDC1, and Ubi were cloned into the lentiviral vector pSIN. CHD1 full‐length cDNA and its truncated mutants with HA tag were cloned into the lentiviral vector pLVX (Addgene). shRNA sequence of SOSTDC1, KPNA1, KPNA2, KPNA3, and CHD1 were cloned into lentiviral vector pLKO.1 (Addgene). HA‐tagged SOSTDC1, FLAG‐tagged CHD1, and β‐TrCP were cloned into bacterial expression vector pGEX‐6p‐1 (Addgene). DR‐GFP (HR report vector) and pCBAScel (I‐Scel expression vector) were kind gifts from Dr. Weixing Zhao. FLAG‐KPNA1, FLAG‐KPNA2, FLAG‐KPNA3, FLAG‐KPNA4, FLAG‐KPNA5, and FLAG‐KPNA6 were kind gifts from Dr. Daming Gao. pBigT‐neo (NHEJ report vector) was a gift from Dr. Anyong Xie. A highly efficient lentiviral system was used to generate plasmid DNA. The breast cancer cell lines were then infected with the lentiviruses to establish stable cell lines. The bacterial expression plasmid was transduced into BL21 *E. coli* bacteria to produce protein. The primers used for cloning are listed in Table S2 (Supporting Information).

### Total RNA Isolation and qRT‐PCR

Total RNA was extracted from cells using TRIzol reagent (Takara Bio) and then reverse‐transcribed to complementary DNA (cDNA) using the HiScript II 1st Strand cDNA Synthesis kit (Vazyme Biotech) according to the manufacture's recommendation. qRT‐PCR was performed to detect the expression levels of target genes using AceQ qPCR SYBR Green Master Mix (Vazyme Biotech) in 7300 Plus Real‐Time PCR System (Applied Biosystems). The relative mRNA level of the target gene was analyzed by the Equation (2)^–Δ^ *^Ct^* (Δ*Ct* = *Ct* of target gene minus Ct of TBP). The primers used are shown in Table S3 (Supporting Information).

### Western Blotting and Separation of Nuclear and Cytoplasmic Proteins

Protein samples were extracted from cells or tissues using RIPA buffer (Beyotime Biotechnology). After determining the concentration by a BCA kit (Thermo Fisher), the protein lysates were denatured in 5× loading buffer in 100 °C for 10 min, separated by SDS‐PAGE, and transferred onto PVDF membranes (Millipore). The membranes were blocked with 5% de‐fat milk, incubated with primary antibody overnight at 4 °C, and probed with HRP‐conjugated secondary antibody. Chemiluminescence detection was carried out with an ImageQuant LAS 4000 mini‐imaging system (GE) with western HRP Substrate (Millipore). Nuclear and Cytoplasmic Protein Extraction Kit (P0028, Beyotime) was used to separate cytoplasmic and nuclear protein according to the manufacture's instructions. Phenylmethylsulfonyl fluoride (PMSF) (ST506, Beyotime) was added to inhibit protein degradation. ImageJ was used to quantify the expression levels of related proteins, which were presented as the ratio of test protein‐integrated density to control protein‐integrated density.

The following antibodies and dilutions were used:

SOSTDC1 (1:200, AF7034, R&D systems), CHD1 (1:1000, A7883, Abclonal), β‐TrCP (1:1000, A1656, Abclonal), γH2AX (1:1000, 60566, CST), Ub (1:500, Santa Cruz Biotechnology), Lamin A/C (1:1000, 4777, CST), Tubulin (1:2000, HC101, TransGen), FLAG (1:2000, F7425, Sigma‐Aldrich), HA (1:1000, C29F4, CST), goat anti‐mouse immunoglobulin G (IgG)–horseradish peroxidase (HRP) (1:5000, HS201‐01, TransGen), goat anti‐rabbit IgG‐HRP (1:5000, HS101‐01, TransGen), donkey anti‐Goat IgG‐HRP (1:5000, SA00001‐3, Proteintech).

### Flow Cytometry Analysis and Sorting

The ALDEFLUOR assay (Stem Cell Technologies) was performed following the manufacturer's protocols. A CD24/CD44 assay was performed with anti‐CD24‐APC (Biolegend,1:40) and anti‐CD44‐APC‐H7 (BD Bioscience,1:100). PE‐conjugated anti‐mouse H2kd antibody (Biolegend, 1:100) was used to discriminate human breast tumor cells from mouse cells in single‐cell suspensions digested by collagenase from xenografted tumors. Four populations from the total tumor cells (ALDH^+^CD24^−^CD44^+^, ALDH^+^non‐CD24^−^CD44^+^, ALDH^−^CD24^−^CD44^+^ and ALDH^−^non‐CD24^−^CD44^+^) were sorted according to the strategy reported previously.^[^ ^30^ ^]^ The cells were collected and divided into two populations (BTICs (ALDH^+^CD24^−^CD44^+^)) and the rest non‐BTICs (ALDH^+^non‐CD24^−^CD44^+^, ALDH^−^CD24^−^CD44^+^, ALDH^−^non‐CD24^−^CD44^+^)) for further analysis. Flow cytometry analysis and cell sorting were conducted by MOFLO ASTRIOS (Beckman Coulter) instrument and analyzed by Summit 6.3 software.

### Immunohistochemistry

The breast cancer patient tumor tissues and para‐tumor tissues were obtained from Shanghai Cancer Hospital affiliated with Fudan University. Informed consent was obtained from the involved patients, and the study was approved by the institution's ethics committee. The tumor tissues of the mouse were fixed in formalin and processed for paraffin embedding. Section samples were dewaxed in xylene and rehydrated in graded alcohol. The immunohistochemistry and signal evaluation (H‐score) were performed according to the procedures reported previously.^[^ ^64^ ^]^

The following antibodies and dilutions were used: SOSTDC1 (1:100, AF7034, R&D systems), Ki67 (1:100, ZM‐0166, ZSGB‐BIO) and peroxidase‐conjugated secondary antibody (KIT‐5010, KIT‐5107, Maxvision).

### MTT Assay

To evaluate the cell proliferation, exponentially growing cells were seeded in 96‐well culture plates (200 to 800 cells per well) and cultured for 3, 5, or 7 days. To determine the cell viability, exponentially growing cells were seeded in 96‐well culture plates (2000 to 10000 cells per well) and incubated overnight to facilitate cell attachment. The following day, the cell cultures were treated with Olaparib (HY‐10162, MCE) in concentrations ranging from 0.0001 to 200 µm. After 72 h, MTT (Biosharp Life Science) was added to each well (final concentration: 0.5 mg mL^−1^) and incubated at 37 °C for 4 h. Then the supernatant was removed, and 100 µL DMSO was added. The optical density value was measured at 490 nm after shaking plates for 10 min. Cell viability was set as 100% for cells not treated with Olaparib.

### Colony Formation Assay

Breast cancer cells were seeded in 6‐well culture plates (200 to 800 cells per well) and cultured at 37 °C for 14 days to allow colonies to form. Cells were fixed with 4% formaldehyde for 30 min and stained with crystal violet. After washing, the cell colonies were quantified.

### Mammosphere Formation Assay

Breast cancer cells were seeded in ultra‐low attachment 96‐well plates (Corning) (100 to 200 cells per well) and cultured with a MammoCult Human Medium kit (StemCell Technologies) supplemented with 4 µg mL^−1^ heparin (StemCell Technologies), 1% pen‐strep, 1 µg mL^−1^ hydrocortisone for 10–14 days. Fresh complete medium was added every four days. The images of mammospheres were collected by microscope for further statistical analysis.

### Immunofluorescence Staining and Confocal Imaging

Breast cancer cells were seeded in 4‐well chamber (Thermo Scientific) (30 000 cells per well) and cultured for 2 days. Cells were fixed with 4% paraformaldehyde (Sangon Biotech) for 15 min at room temperature (RT), membrane perforated with 0.15% Triton X‐100 (Sangon Biotech) for 10 min at RT, blocked with 5% bovine serum albumin (Sangon Biotech) solution for 1 h at RT, incubated with primary antibody overnight at 4 °C and secondary antibody for 40 min at RT. The cell nucleus was stained with DAPI (Invitrogen). Images were captured by confocal microscope (LEICA SP5) with a 63× objective lens.

The following antibodies and dilutions were used: SOSTDC1 (1:20, AF7034, R&D systems), CHD1 (1:100, A7883, Abclonal), FLAG (1:100, F7425, Sigma–Aldrich), γH2AX (1:100, 60566, CST), goat anti‐rabbit IgG secondary antibody Alexa Fluor 546 (1:100, A‐11035, Invitrogen), donkey anti‐goat IgG secondary antibody Alexa Fluor 488 (1:100, A‐11055, Invitrogen).

### HR and NHEJ Reporter Assay

293T cells integrated with DR‐GFP or pBigT‐neo‐GFP cassettes were used to determine HR or NHEJ efficiency, respectively. Cells transiently transfected with report vectors or control‐mcherry vector, control or SOSTDC1‐overexpressing plasmids were then transfected with I‐Scel expression vector pCBAScel. 36 h after transfection, the percentage of GFP^+^ or mCherry^+^ cells was analyzed by flow cytometry. HR or NHEJ efficiency was presented as the percentage of GFP^+^/mCherry^+^. HR or NHEJ efficiency presented in the figures are the mean ± SEM of three independent experiments.

### Chemical Inhibitors

PARP inhibitor Olaparib (HY‐10162, MCE), treated breast cancer cells at concentrations of 0.1 nmol L^−1^‐200 µmol L^−1^ in vitro and treated xenograft tumors (50 mg kg^−1^, once a day) for 29 days intraperitoneally in vivo. Eukaryote protein synthesis inhibitor CHX (HY‐12320, MCE), treated breast cancer cells at a concentration of 100 µg mL^−1^. Proteasome inhibitor MG132 (HY‐13259, MCE), treated breast cancer cells at a concentration of 10 µmol L^−1^.

### Immunoprecipitation Assay and Ubiquitylation

Breast cancer cells were collected and lysed by the mild RIPA lysis containing protease inhibitor cocktail (Roche) for 1 h at 4 °C. Except for a small fraction of input group, the remaining supernatant was diluted with the week RIPA lysis and incubated with magnetic FLAG‐Beads (Sigma–Aldrich) or anti‐HA antibody‐conjugated protein A/G agarose beads (Smart‐Lifesciences) overnight at 4 °C. After removing the supernatant, beads were washed 4 times with NETN buffer (20 mmol L^−1^ Tris, 100 mmol L^−1^ NaCl, 0.5% NP40, 1 mmol L^−1^ EDTA, pH8.0). Proteins were then eluted by competitive elution of 3× FLAG fusion proteins (0.4 mg ml^−1^; F4799, Sigma‐Aldrich) and boiled or directly denatured in 2× loading buffer. Boiled samples can be followed by silver staining and western blotting. To examine the ubiquitylation level of CHD1, cells were treated with MG132 (10 µmol L^−1^, 8 h, MCE) before harvested. Protein lysates were performed with immunoprecipitation and western blotting.

### Protein Purification and GST Pull‐Down Assay

BL21 *E. coli* bacteria were transfected with GST‐tagged vectors and grown in Luria–Bertani liquid medium to an OD600 of ≈0.8, and then adding with isopropyl β‐d‐1‐thiogalactopyranoside (IPTG, 1 mmol L^−1^) to induce the protein expression overnight at 16 °C. Proteins were purified using the GST agarose beads (TransGen Biotech) from the cell lysates and then eluted with 10 mmol L^−1^ GSH (BBI). Samples were subject to co‐IP assay or western blotting.

### In Vitro Ubiquitination Assay

GST‐fusion proteins (β‐TrCP, FLAG‐tagged CHD1, HA‐tagged SOSTDC1) were purified from BL21 strain and added into the ubiquitination buffer system (10x reaction buffer, 10x E1 enzyme, 5x Ubiquitin, 20x UBE2D3/UbcH5c, 10x Mg2^+^‐ATP) provided by E2Select Ubiquitin Conjugation Kit (ubbiotech). The reaction was conducted at 37 °C for 3 h and then mixed with loading buffer subjected to following western blotting.

### Mass Spectrometry

The co‐IP protein samples of SUM159‐CTRL or SOSTDC1 and MDA‐MB‐231‐CTRL or SOSTDC1 cells were concentrated in stacking gel. Gel‐containing samples were decolorized and washed to make it transparent and then freeze‐dried. Samples were reduced of disulfide bonds before enzymatic hydrolysis. Then, the peptide segment was extracted and dried in a vacuum. Samples were desalted, and the supernatant was added to the sample bottle for mass spectrometry (Q Exactive) detection. The search database was Maxquant.

### RNA Sequencing

After RNA extraction, RNA concentration and quality were measured by Agilent 2100. RNA‐seq libraries were established and sequenced on HiSeq3000 platform (Illumina). For analyzing the RNA‐sequencing results, the differential gene expression was determined by DEseq using 1.5‐fold change, with *p*‐value < 0.05 as threshold.

### Quantification and Statistical Analysis

All experiments for quantitative analysis and representative images were reproduced with similar results for at least three times. Bar graphs were generated with GraphPad Prism 6.0, and all values are reported as the means ± SEM. Comparisons between two groups were performed using an unpaired Student's *t*‐test or Mann–Whitney U‐test. Differences among three or more groups were analyzed by one‐way/two‐way ANOVA. Two‐sided log‐rank (Mentel–Cox) test was used to evaluate the survival analysis. Pearson Chi‐square test was used to evaluate IHC score levels between different clinicopathological variable groups. Bivariate correlation analysis was performed using the Pearson correlation method. *p* < 0.05 was considered statistically significant.

### Public Data Analysis

GSE165914 for Olaparib‐resistant gene cluster^[^ ^44^ ^]^ was obtained from Gene Expression Omnibus of the National Center for Biotechnology Information (NCBI) (https://www.ncbi.nlm.nih.gov/geo). PRJNA739366 and PRJNA376644 data were obtained from the Sequence ReadArchive of NCBI. Results were obtained with the online tool bc‐GenExMiner v4.5, for which the available URL is http://bcgenex.ico.unicancer.fr/BC‐GEM/GEM‐requete.php. Kaplan–Meier plotter was used to analyze the OS and RFS of different molecular subtypes of breast cancer patients based on SOSTDC1 expression. A total of 1220 BC patients from The Cancer Genome Atlas (https://tcga‐data.nci.nih.gov/tcga) were used for gene expression analysis for SOSTDC1 and CHD1. The NLS sequence was analyzed by NLS Mapper (http://nls‐mapper.iab.keio.ac.jp/cgi‐bin/NLS_Mapper_form.cgi).

### Ethical Statement

The mice experiments were conducted according to standard operating procedures in accordance with the recommendations of the Guide for the Care and Use of Laboratory Animals of Fudan University, and approved by the Care and Use of Laboratory Animal of Fudan University (202401FD0003). The breast cancer patient tumor tissues and para‐tumor tissues were obtained from Shanghai Cancer Hospital affiliated with Fudan University. Informed consent was obtained from the involved patients, and the study was approved by the institution's ethics committee (Fudan University Shanghai Cancer Center Institutional Review Board, 050432‐4‐2108*).

## Conflict of Interest

The authors declare no conflict of interest.

## Author Contributions

Q.D.D. and J.K.Q. contributed equally to this work. Q.D.D., J.K.Q., L.X.Z., and S.L.L performed conceptualization. Q.D.D., J.K.Q. performed investigation. Q.D.D., J.K.Q., C.C.L., J.H.X., L.X.Z. W.M., L.Z. J.X., X.L.P., S.Q.L., X.C., Y.Z.J., Z.M.S., C.S.C did Methodology. Q.D.D., J.K.Q., C.C.L. visualized the study. Q.D.D., J.K.Q. wrote the original draft. Q.D.D., J.K.Q., S.L.L., J.H.X., L.X.Z. wrote, reviewed, and edited the final report. S.L.L., J.H.X., L.X.Z. supervised the study. L.X.Z., S.L.L. acquired funding.

## Supporting information

Supporting Information

## Data Availability

The data that support the findings of this study are available from the corresponding author upon reasonable request.
