# Supplementary material for: SOSTDC1 Nuclear Translocation Facilitates BTIC Maintenance and CHD1‐Mediated HR Repair to Promote Tumor Progression and Olaparib Resistance in TNBC
Source: Adv Sci (Weinh). 2024 Jun 12;11(29):2306860. doi: 10.1002/advs.202306860 (PMC11304230; doi:10.1002/advs.202306860)
Supplement: Supplementary file 1 — Supporting Information [file ADVS-11-2306860-s001.docx]

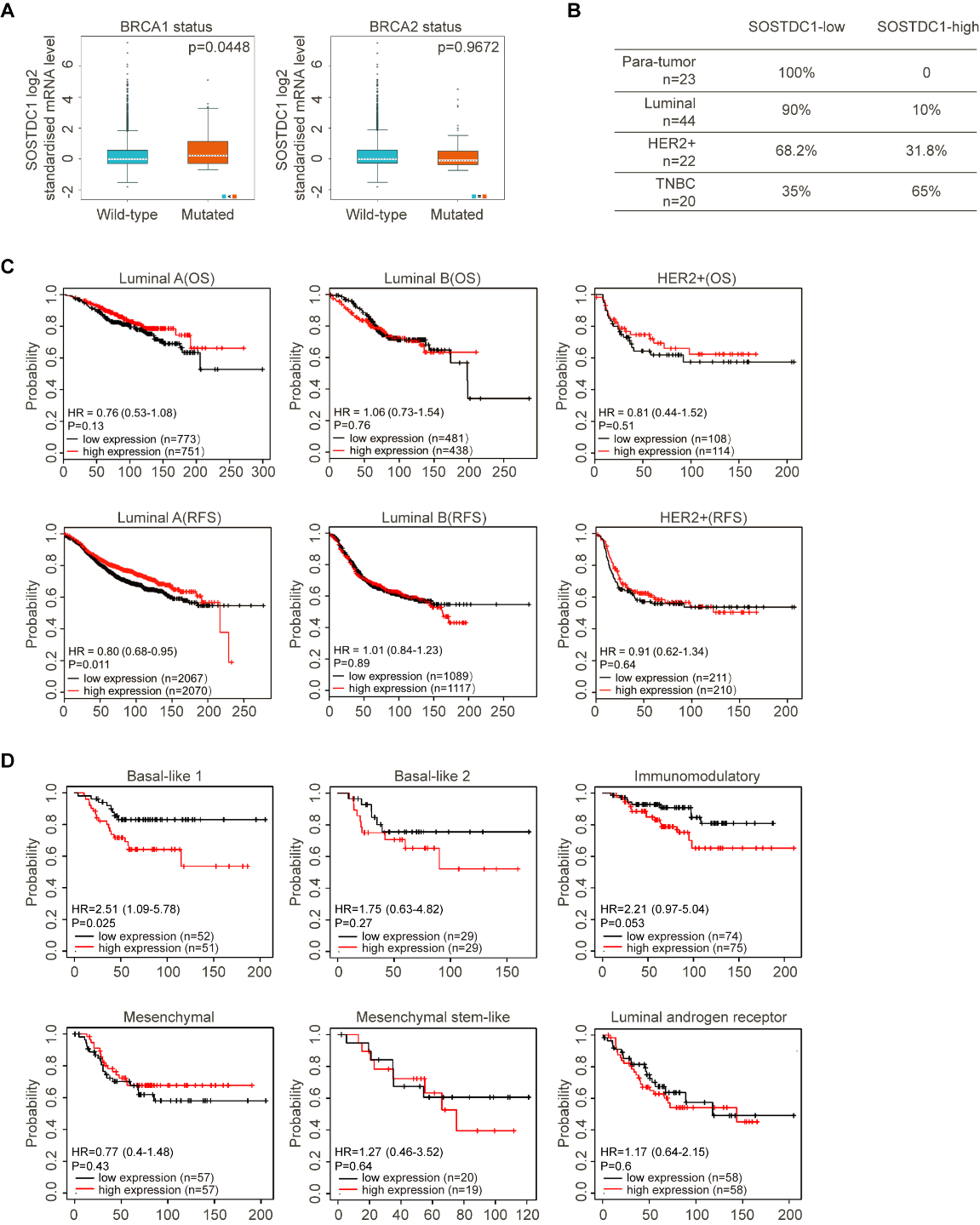


**Figure S1. SOSTDC1 is highly expressed in TNBC and indicates a poor prognosis.** (A) The mRNA expression levels of SOSTDC1 were analyzed in BRCA1 or BRCA2 mutated (n=72, n=56) or wildtype (n=2116, n=2130) breast tumors (http://bcgenex.ico.unicancer.fr/BC-GEM/GEM-requete.php). (B) Percentage of SOSTDC1 low-expressing (H-Score<20) and high-expressing (H-Score>20) tissues in different subtypes of BC tissues and para-tumor tissues according to IHC staining in Figure 1.C. (C) Overall survival and relapse-free survival of Luminal A, Luminal B, HER2+ subtypes of breast cancer patients in Kaplan-Meier Plotter with high or low SOSTDC1 expression. Statistical significance was assessed using a log-rank test. (D) Overall survival of Basal-like 1, Basal-like 2, Immunomodulatory, Mesenchymal, Mesenchymal stem–like, Luminal androgen receptor subtypes of TNBC patients in Kaplan-Meier Plotter with high or low SOSTDC1 expression. Statistical significance was assessed using a log-rank test.


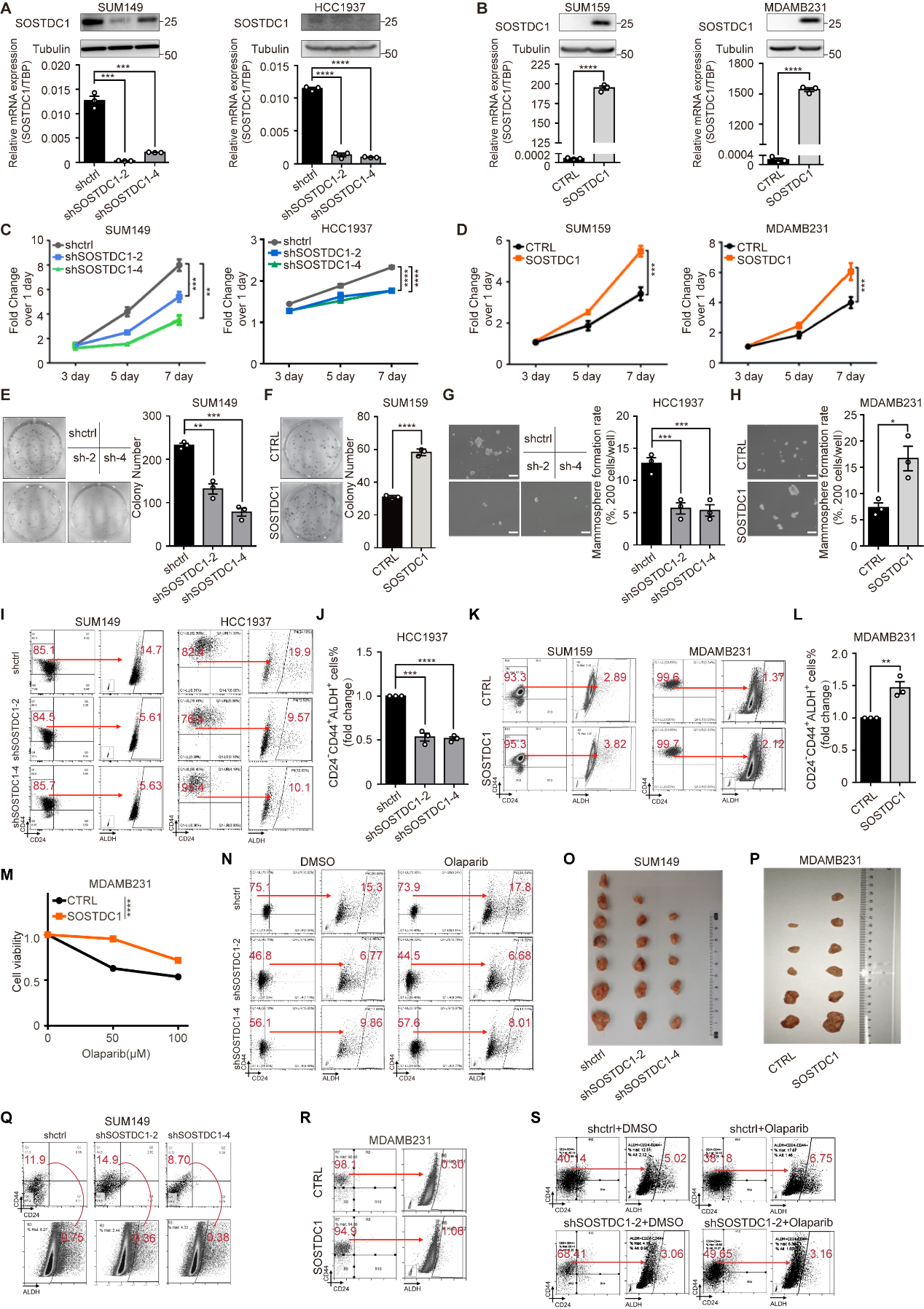


**Figure S2. SOSTDC1 is positively correlated with the TNBC malignancy.** (A-B) qRT-PCR (bottom) and western blotting (top) were used to verify the knockdown efficiency of SOSTDC1 in SUM149 and HCC1937 cells (A) and the overexpressing efficiency of SOSTDC1 in SUM159 and MDA-MB-231 cells (B). Data were presented as mean ± SEM. Statistical significance was determined using the one-way ANOVA or unpaired Student’s two-sided t-tests. ***p < 0.001, ****p < 0.0001. (C-D) MTT assays of cell proliferation ability in SOSTDC1-knockdown SUM149 or HCC1937 cells (C) or SOSTDC1-overexpressing SUM159 or MDA-MB-231 cells (D). n = 6 independent biological samples. (E-F) Plate colony formation assay of SOSTDC1-knockdown SUM149 cells (E) or SOSTDC1-overexpressing SUM159 cells (F). Colonies in the whole field were counted and data were presented as mean ± SEM, following the one-way ANOVA or the unpaired Student’s two-sided t-test. **p < 0.01, ***p < 0.001, ****p < 0.0001. (G-H) Mammosphere formation assays of SOSTDC1-knockdown HCC1937 cells (G) or SOSTDC1-overexpressing MDA-MB-231 cells (H). Representative images were shown and data were presented as mean ± SEM, following the one-way ANOVA or the unpaired Student’s two-sided t-test. *p < 0.05, ***p < 0.001. Scale bar, 100μm. (I) Representative flow cytometry analysis results of BTIC population in SOSTDC1-knockdown SUM149 or HCC1937 cells. (J) The percentage of the BTIC population of SOSTDC1-knockdown HCC1937 cells. Data were presented as mean ± SEM. Statistical analysis was performed using the one-way ANOVA. ***p < 0.001, ****p < 0.0001. (K) Representative flow cytometry analysis results of BTIC population in SOSTDC1-overexpressing SUM159 or MDA-MB-231 cells. (L) The percentage of the BTIC population of SOSTDC1-overexpressing MDA-MB-231 cells. Data were presented as mean ± SEM. Statistical analysis was performed using the unpaired Student’s two-sided t-test. **p < 0.01. (M) Cell viability determined by MTT assays in SOSTDC1-overexpressing MDA-MB-231 cells with Olaparib treatment at the indicated concentrations. n = 3 independent biological samples. (N) Representative flow cytometry analysis results of BTIC population in SOSTDC1-knockdown SUM149 cells treated with 10 nmol L^−1^ Olaparib for 5d. (O) Analysis of SOSTDC1-knockμdown SUμ149 xenografts growth in vivo. A representative picture of the tumors was shown. (P) Analysis of SOSTDC1-overexpressing MDA-MB-231 xenografts growth in vivo. A representative picture of the tumors was shown. (Q) Representative flow cytometry analysis results of BTIC population in SOSTDC1-knockdown SUM149 xenografts. (R) Representative flow cytometry analysis results of BTIC population in SOSTDC1-overexpressing MDA-MB-231 xenografts. (S) Representative flow cytometry analysis results of BTIC population in SOSTDC1-knockdown SUM149 xenografts treated with DMSO or Olaparib.


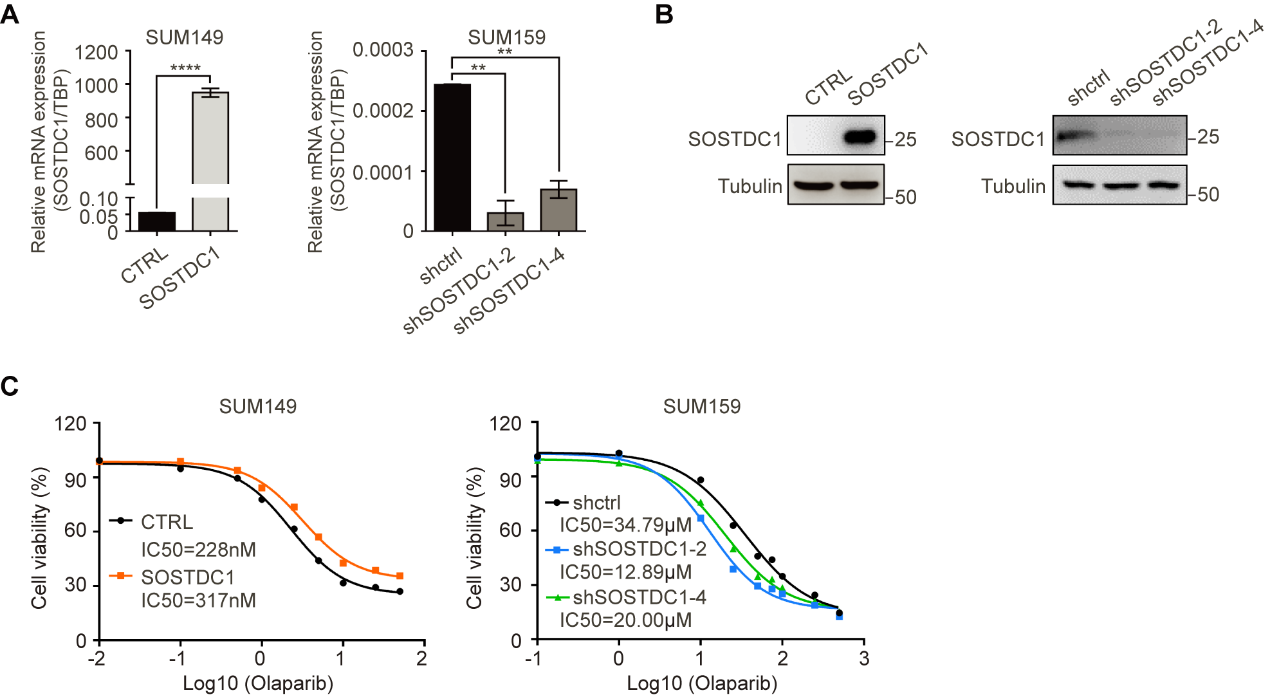


**Figure S3. SOSTDC1 expression is correlated with cell response to Olaparib in TNBC cells.** (A) qRT-PCR were used to verify the overexpression and knockdown efficiency of SOSTDC1 in SUM149 and SUM159 cells. (B) Western blotting analysis were used to verify the overexpression and knockdown efficiency of SOSTDC1 in SUM149 and SUM159 cells. (C) Cell viability determined by MTT assays in SOSTDC1-overexpressing SUM149 cells and SOSTDC1-knockdown SUM159 cells with Olaparib treatment at the indicated concentrations. n = 3 independent biological samples.


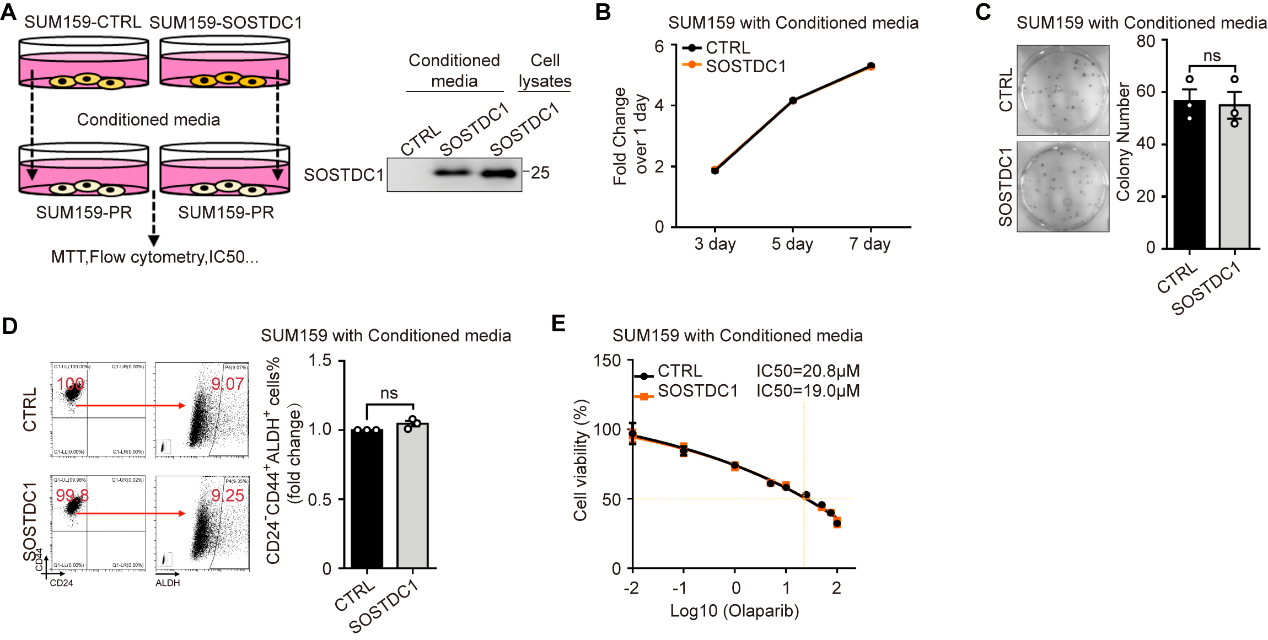


**Figure S4. The malignant functions of SOSTDC1 are independent on its** **secreted form.** (A) Schematic diagram of treating the parental SUM159 cells with conditioned media from SOSTDC1-overexpressing SUM159 cells. Western blotting analysis of SOSTDC1 in the lysate or media (secreted). (B) MTT assays of cell proliferation ability in parental SUM159 cells treated with conditioned media from SOSTDC1-overexpressing SUM159 cells. n = 6 independent biological samples. **(**C) Plate colony formation assay of parental SUM159 cells treated with conditioned media from SOSTDC1-overexpressing SUM159 cells. Colonies in the whole field were counted and data were presented as mean ± SEM, following the unpaired Student’s two-sided t-test. ns, not significant. (D) The percentage of the BTIC population of parental SUM159 cells treated with conditioned media from SOSTDC1-overexpressing SUM159 cells. Representative flow cytometry analysis results were shown on the left. Data were presented as mean ± SEM. Statistical analysis was calculated using the unpaired Student’s two-sided t-test. ns, not significant. (E) Cell viability in response to Olaparib was determined by MTT assays in parental SUM159 cells treated with conditioned media from SOSTDC1-overexpressing SUM159 cells. n = 3 independent biological samples.


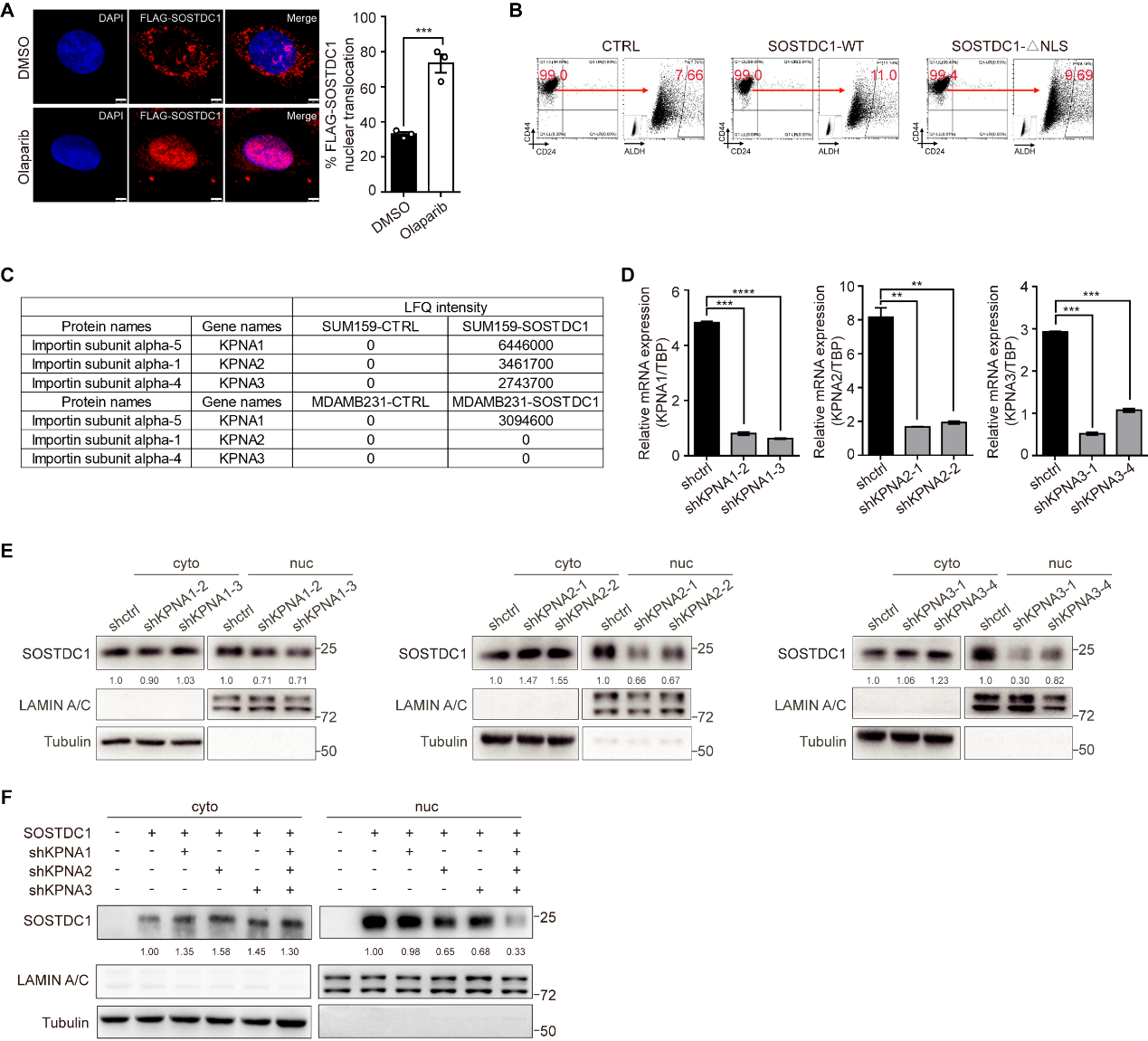


**Figure S5. SOSTDC1 translocates to the nucleus in an importin-α dependent manner.** (A) SOSTDC1-overexpressing U2OS cells were treated with DMSO or 5μmol L^−1^ Olaparib for 12h before immunofluorescence analysis using anti-FLAG antibody. Representative images were shown and percentages of SOSTDC1 nuclear translocation were calculated. Data were presented as mean ± SEM, following the unpaired Student’s two-sided t-test. ***p < 0.001. Scale bar, 5μm. (B) Representative flow cytometry analysis results of BTIC population in wild type (WT) or NLS-deleted mutant (ΔNLS) SOSTDC1-overexpressing SUM159 cells. (C) Analysis of KPNA1, KPNA2, KPNA3 intensity in mass spectrometry results of anti–FLAG-SOSTDC1 co-IP protein samples. (D) qRT-PCR was used to verify the knockdown efficiency of KPNA1, KPNA2, KPNA3 in SOSTDC1-overexpressing SUM159 cells. Data were presented as mean ± SEM, following the one-way ANOVA. **p < 0.01, ***p < 0.001, ****p < 0.0001. (E) Analysis of the expression of cytoplasmic and nuclear SOSTDC1 after 1μmol L^−1^ Olaparib treatment for 12h by western blotting in SOSTDC1-overexpressing SUM159 cells with KPNA1 or KPNA2 or KPNA3 knockdown. (F) Analysis of the expression of cytoplasmic and nuclear SOSTDC1 by western blotting in SOSTDC1-overexpressing SUM159 cells with KPNA1, KPNA2, KPNA3 knockdown separately or simultaneously.


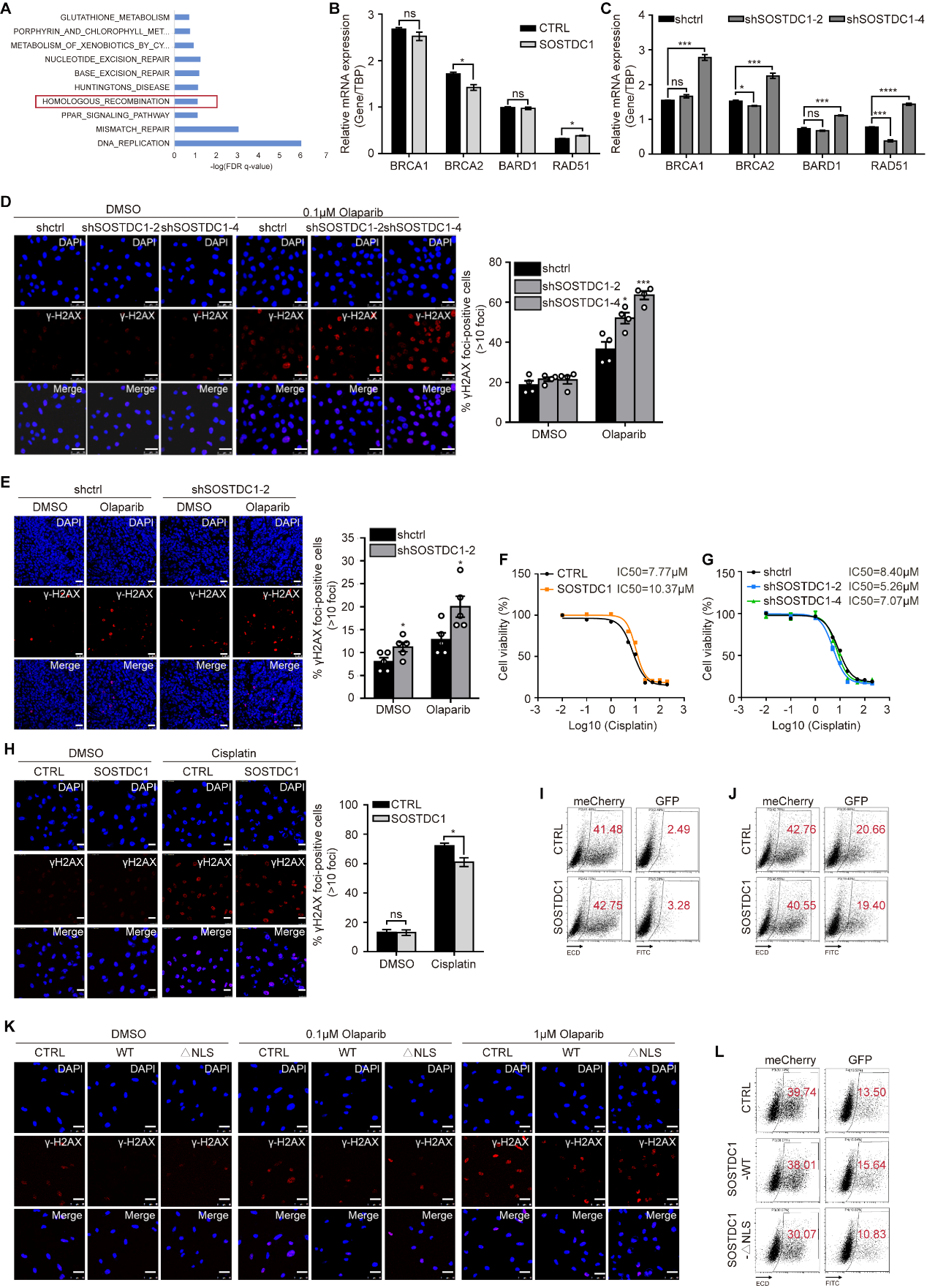


**Figure S6. Nuclear SOSTDC1 promotes HR repair.** (A) KEGG pathway analysis showed the enrichment for HR repair-related genes with increased expression in SOSTDC1-overexpressing SUM159 cells. (B-C) mRNA expression levels of BRCA1, BRCA2, BARD1, RAD51 in SOSTDC1-overexpressing SUM159 cells (B) or SOSTDC1-knockdown SUM159 cells (C). (D) Immunofluorescence analysis of γ-H2AX foci in SOSTDC1-knockdown SUM149 cells treated with DMSO or 0.1μmol L^−1^ Olaparib for 12h. Representative images were shown and percentages of γ-H2AX foci-positive cells (>10 foci) were quantified (100 cells each experiment). Data were presented as mean ± SEM. Statistical analysis was performed using the one-way ANOVA. *p < 0.05, ***p < 0.001. Scale bar, 50μm. (E) Immunofluorescence analysis of γ-H2AX foci in SOSTDC1-knockdown SUM149 xenografts treated with DMSO or Olaparib. Representative images were shown and percentages of γ-H2AX foci-positive cells (>10 foci) were quantified (250 cells each experiment). Data were presented as mean ± SEM, following unpaired Student’s two-sided t-tests. Scale bar, 25μm. (F-G) Cell viability determined by MTT assays in SOSTDC1-overexpressing SUM159 cells (F) and SOSTDC1-knockdown SUM159 cells (G) with Cisplatin treatment at the indicated concentrations. n = 3 independent biological samples. (H) Immunofluorescence analysis of γ-H2AX foci in SOSTDC1-overexpressing SUM159 cells treated with DMSO or 5μmol L^−1^ Cisplatin for 12h. Representative images were shown and percentages of γ-H2AX foci-positive cells (>10 foci) were quantified (100 cells each experiment). Data were presented as mean ± SEM. Statistical analysis was performed using the one-way ANOVA. *p < 0.05, ns, not significant. Scale bar, 25μm. (I) Representative flow cytometry analysis results of HR efficiency in SOSTDC1-overexpressing cells using the DR-GFP reporter assay. (J) Representative flow cytometry analysis results of NHEJ efficiency in SOSTDC1-overexpressing cells using the pBigT-neo-GFP reporter assay. (K) Immunofluorescence analysis of γ-H2AX foci in wild type (WT) or NLS-deleted mutant (ΔNLS) SOSTDC1-overexpressing SUM159 cells treated with DMSO or Olaparib (0.1μmol L^−1^, 1μmol L^−1^) for 12h. Representative images were shown. Scale bar, 50μm. (L) Representative flow cytometry analysis results of HR efficiency in wild type (WT) or NLS-deleted mutant (ΔNLS) SOSTDC1-overexpressing SUM159 cells using the DR-GFP reporter assay.


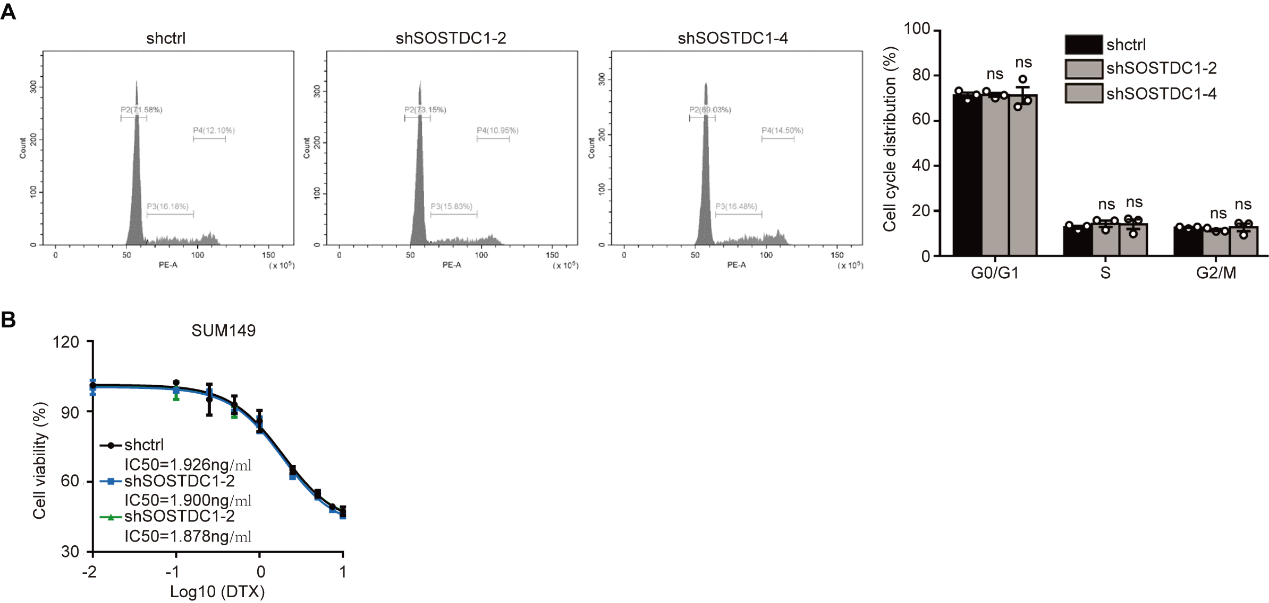


**Figure S7. SOSTDC1 knockdown did not affect cell-cycle distribution and cell sensibility to chemotherapy drug Docetaxel.** (A) Cell cycle distribution of SOSTDC1-knockdown SUM149 cells. Data were presented as mean ± SEM, following the one-way ANOVA. ns, not significant. (B) Survival analysis of SOSTDC1-knockdown SUM149 cells in response to DTX; n = 3 independent biological samples.


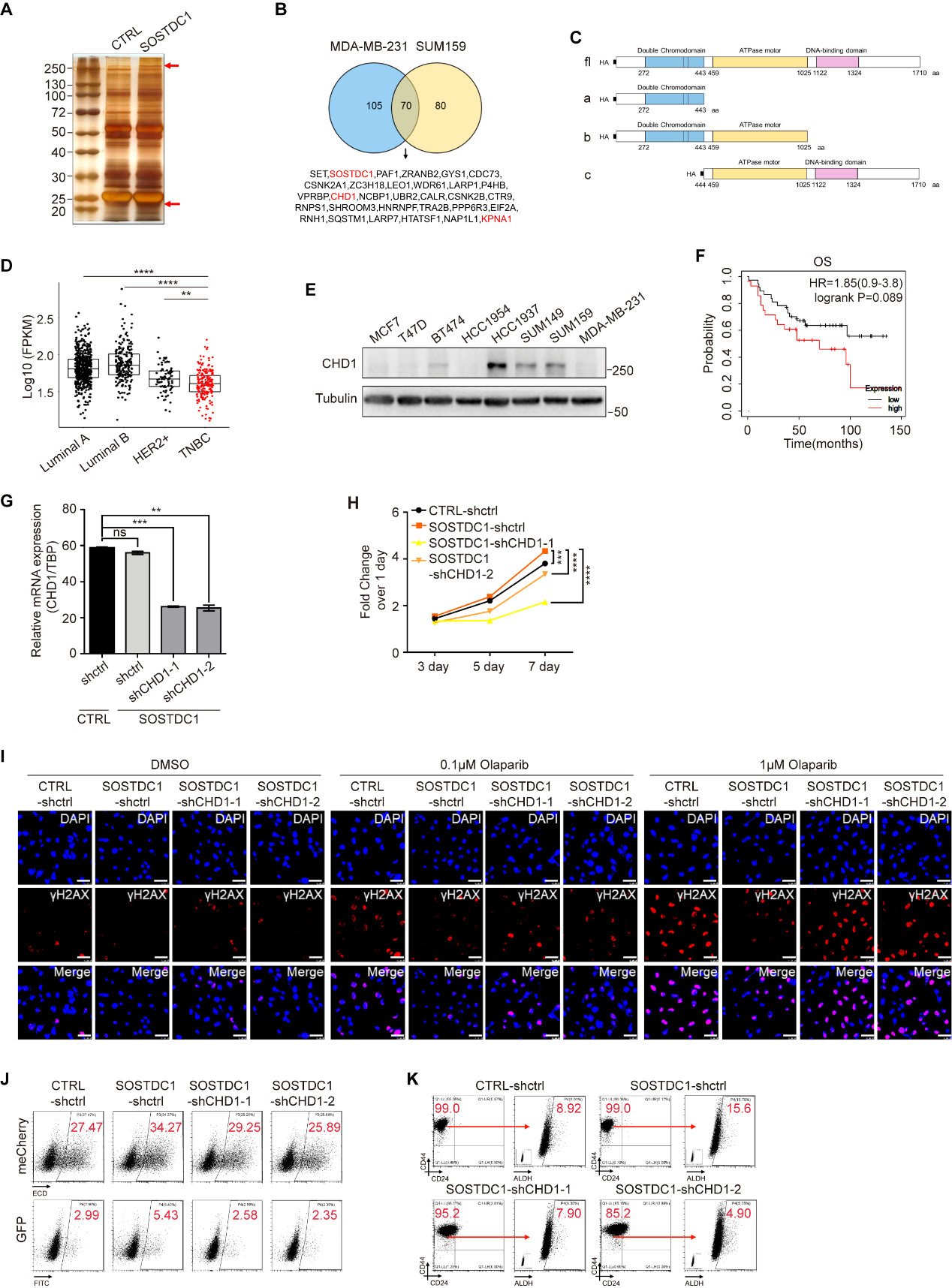


**Figure S8. CHD1 protein is highly expressed in TNBC and is necessary to SOSTDC1 function.** (A) Representative image of silver staining after co-IP in SOSTDC1-overexpressing MDA-MB-231 cells. The bottom red arrowhead, ~25kD. The top red arrowhead, ~255kD. (B) Mass spectrometry analysis of the co-IP samples (control and SOSTDC1-overexpreing) indicated candidate proteins interacted with SOSTDC1. (C) Schematic diagram of CHD1 in different truncated forms. (D) CHD1 mRNA expression levels of patients in the TCGA database with different molecular subtypes of breast cancer. Statistical significance was determined using Mann–Whitney U-tests. **p < 0.01, ****p < 0.0001. (E) CHD1 protein expression in different subtypes of BC cell lines. (F) Overall survival of BC patients in Kaplan-Meier Plotter with high or low CHD1 protein expression. Statistical significance was assessed using a log-rank test. (G) qRT-PCR was used to verify the knockdown efficiency of CHD1 in SOSTDC1-overexpressing SUM159 cells. Data were presented as mean ± SEM, following the one-way ANOVA. **p < 0.01, ***p < 0.001. ns, not significant. (H) MTT assays of cell proliferation ability in SOSTDC1-overexpressing SUM159 cells with CHD1-knockdown. n = 6 independent biological samples. (I) SOSTDC1-overexpressing SUM159 cells with CHD1-knockdown were treated with DMSO or Olaparib (0.1μmol L^−1^, 1μmol L^−1^) for 12h, following immunofluorescence analysis of γ-H2AX foci. Representative images were shown. Scale bar, 50μm. (J) Representative flow cytometry analysis results of HR efficiency in SOSTDC1-overexpressing SUM159 cells with CHD1-knockdown using the DR-GFP reporter assay. (K) Representative flow cytometry analysis results of BTIC population in SOSTDC1-overexpressing SUM159 cells with CHD1-knockdown.


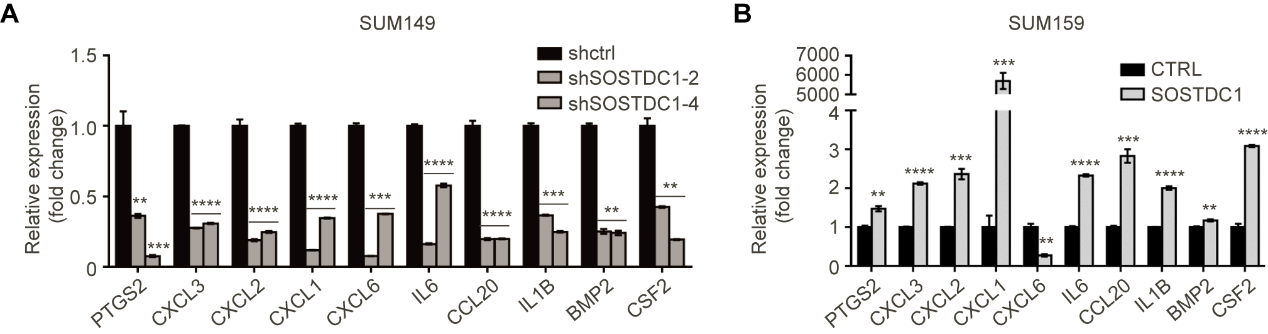


**Figure S9. SOSTDC1 promotes CHD1-regulating gene expression.** (A-B) CHD1-regulating gene expression levels in SOSTDC1-knockdown SUM149 cells (A) or SOSTDC1-overexpressing SUM159 cells (B). Data were presented as mean ± SEM. Statistical significance was performed using the one-way ANOVA or unpaired Student’s two-sided t-tests. **p < 0.01, ***p < 0.001, ****p < 0.0001.


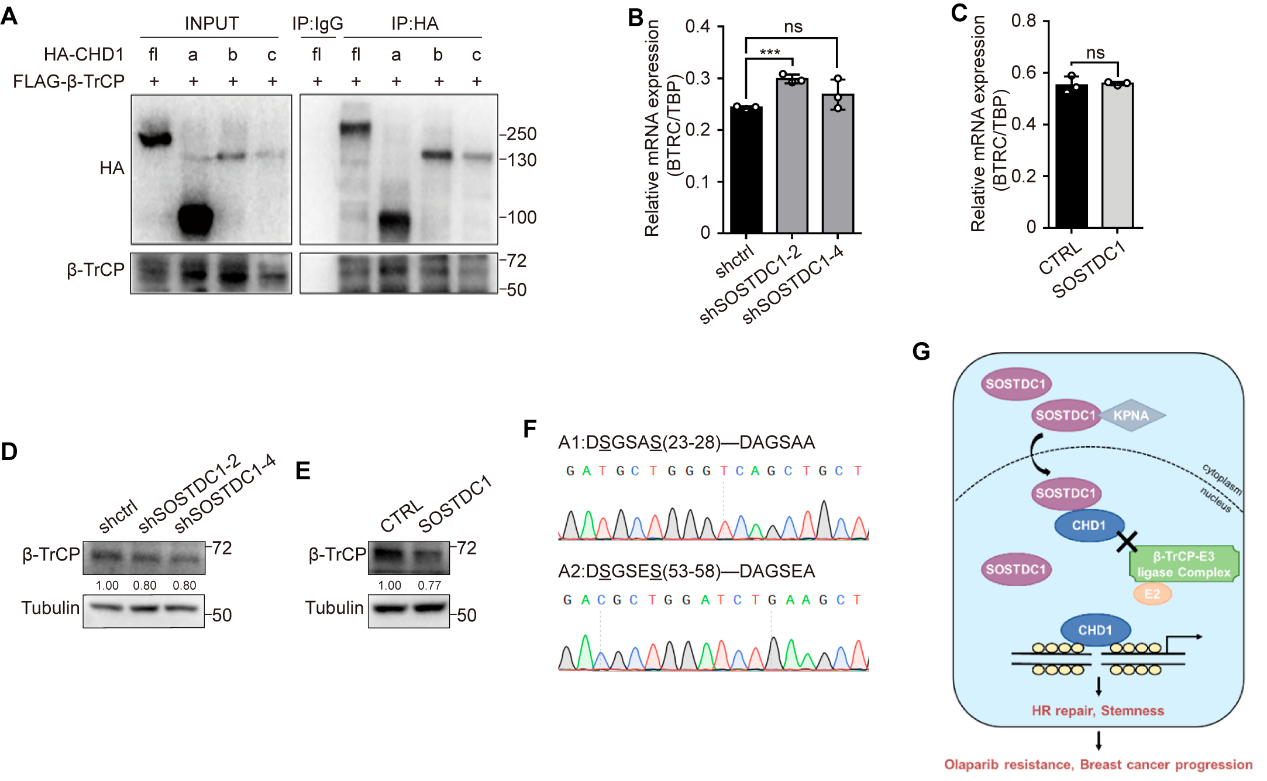


**Figure S10. SOSTDC1 inhibits β-TrCP-mediated CHD1 ubiquitination by blocking the interaction of β-TrCP with CHD1.** (A) Co-IP assay of the putative interaction between β-TrCP and full length CHD1 (fl) or its three truncated mutants (a, b, c). (B-C) β-TrCP mRNA expression in SOSTDC1-knockdown SUM149 cells (B) or SOSTDC1-overexpressing SUM159 cells (C). Data were presented as mean ± SEM. Statistical significance was performed using the one-way ANOVA or the unpaired Student’s two-sided t-test. ***p < 0.001. ns, not significant. (D-E) β-TrCP protein expression in SOSTDC1-knockdown SUM149 cells (D) or SOSTDC1-overexpressing SUM159 cells (E). (F) Mutant sequences of two β-TrCP binding motif of CHD1. A1, mutant of serine 24,28 replaced by alanine; A2, mutant of serine 54,58 replaced by alanine. (G) Model for SOSTDC1 nuclear translocation mediated Olaparib resistance and TNBC progression.

|  |  |  | SOSTDC1 expression | | |  |  |
| --- | --- | --- | --- | --- | --- | --- | --- |
| Variables | Number of patients N [%] |  | Low N [%] |  | High N [%] |  | P^a)^ value |
|  |  |  |  |  |  |  |  |
| **Total** | 86 |  | 62(72.1)) |  | 24(27.9) |  |  |
| **Age** |  |  |  |  |  |  |  |
| <=50 | 26(30.2) |  | 22(25.6) |  | 4(4.7) |  | 0.088 |
| >50 | 60(69.8) |  | 40(46.5) |  | 20(23.3) |  |  |
| **Tumor size** |  |  |  |  |  |  |  |
| <=3 | 64(74.4) |  | 43(50) |  | 21(24.4) |  | *0.037 |
| >3 | 21(24.4) |  | 19(22.1) |  | 2(2.3) |  |  |
| unknown | 1(1.2) |  | 0(0) |  | 1(1.2) |  |  |
| **Lymph node status** |  |  |  |  |  |  |  |
| negative | 30(34.9) |  | 22(25.6) |  | 8(9.3) |  | 0.741 |
| positive | 43(50) |  | 30(34.9) |  | 13(15.2) |  |  |
| unknown | 13(15.1) |  | 10(11.6) |  | 3(3.5) |  |  |
| **Grade** |  |  |  |  |  |  |  |
| II | 22(25.9) |  | 20(23.3) |  | 2(2.3) |  | *0.019 |
| III | 56(65.1) |  | 36(41.9) |  | 20(23.3) |  |  |
| unknown | 8(9.3) |  | 6(7.0) |  | 2(2.3) |  |  |
| **ER status** |  |  |  |  |  |  |  |
| negative | 41(47.7) |  | 21(24.4) |  | 20(23.3) |  | ***<0.001 |
| positive | 45(52.3) |  | 41(47.7) |  | 4(4.7) |  |  |
| **PR status** |  |  |  |  |  |  |  |
| negative | 50(58.1) |  | 29(33.7) |  | 21(24.4) |  | *** 0.001 |
| positive | 36(41.9) |  | 33(38.4) |  | 3(3.5) |  |  |
| **HER2/neu status** |  |  |  |  |  |  |  |
| negative | 22(25.6) |  | 11(12.8) |  | 11(12.8) |  | ** 0.007 |
| positive | 64(74.4) |  | 51(59.3) |  | 13(15.1) |  |  |
| **Subtype** |  |  |  |  |  |  |  |
| Luminal | 44(51.2) |  | 40(46.5) |  | 4(4.7) |  | ***<0.001 |
| HER2+ | 22(25.6) |  | 15(17.4) |  | 7(8.1) |  |  |
| TNBC | 20(23.3) |  | 7(8.1) |  | 13(15.1) |  |  |

**Table S1. Clinicopathological variables and expression of SOSTDC1.**

^a^ Based on Pearson χ2 test (Fisher exact test was used when needed)

**Table S2. Primer sequences for plasmid construction.**

| Identifier | Sequence (5'-3') |
| --- | --- |
|  |  |
| SOSTDC1-FLAG-pSIN-F | CAAGGATGACGATGACAAGACGCGTATGCTTCCTCCTGCCATTCA |
| SOSTDC1-FLAG-pSIN-R | GCCGCTCTAGACTACTCGAGCTAACTCATGCTGTGCTT |
| SOSTDC1-ΔNLS-FLAG-pSIN-R1 | TTTGCTGGCTCTTTTCCGGACTACTGTGATTTTGTAGG |
| SOSTDC1-ΔNLS-FLAG-pSIN-F2 | CGGAAAAGAGCCAGCAAATCCAGCAAGCACAGCATGAGTTAG |
| SOSTDC1-ΔNLS-FLAG-pSIN-R2 | CATATGTTCGAAGAATTCCTAACTCATGCTGTGCTTGCTGGATTTGCTGGCTCTTTTCCG |
| SOSTDC1-pSIN-F | GTGAGGAATTGGGATCCGCCACCATGATGCTTCCTCCTGCCATTCATT |
| CHD1-HA-PLVX-F | GATGTTCCAGATTACGCTCTCGAGATGAATGGACACAGTGATGAAGAA |
| CHD1-A-HA-PLVX-R | CGCTCTAGACTACTCGAGTTACCTGCTAAAATACTCATCAATGCA |
| CHD1-B-HA-PLVX-R | GGCCGCTCTAGACTACTCGAGTTATTCAGCTCTCTTCAAGATTTCATCT |
| CHD1-C-HA-PLVX-F | GATGTTCCAGATTACGCTCTCGAGAACCAATCAAAAACCACTCCTTTT |
| CHD1-HA-PLVX-R | GGCCGCTCTAGACTACTCGAGTTATGTTTTCCGACTACTCCAGGTA |
| BTRC-FLAG-pSIN-F | GATGACGATGACAAGACGCGTATGGACCCGGCCGAGGCGGTGC |
| BTRC-FLAG-pSIN-R | TTCATATGTTCGAAGAATTCTTATCTGGAGATGTAGGTGTATGTTCGAG |
| Ub-pSIN-F | GAGGAATTGGGATCCGCCACCATGATGCAGATCTTCGTGAAGACT |
| Ub-pSIN-R | TGTTCGAAGAATTCGTCCGGCCCACCTCTGAGACGGAG |
| CHD1-FLAG-pGEX-F | GGGCCCCTGGGATCCATGAATGGACACAGTGATGAAGA |
| CHD1-FLAG-pGEX-R | TCGAGTCGACCCGGGAATTCTTACTTATCGTCGTCATCCTTGTAATCTGTTTTCCGACTACTCCA |
| SOSTDC1-HA-pGEX-F | TCCAGGGGCCCCTGGGATCCATGCTTCCTCCTGCCATTCA |
| SOSTDC1-HA-pGEX-R | TCGAGTCGACCCGGGAATTCCTAAGCGTAATCTGGAACATCGTATGGGTAGTTAACCATACTCATGCTGTGCTTGCT |
| BTRC-pGEX-F | TCCAGGGGCCCCTGGGATCCATGGACCCGGCCGAGGCGGT |
| BTRC-pGEX-R | TCGAGTCGACCCGGGAATTCTTATCTGGAGATGTAGGTGT |
| shSOSTDC1-F2 | TACTCGAGTATGCGACCGTAAAGAGCTACTTTTTTGAATTCTCGACCTCGAG |
| shSOSTDC1-R2 | CGCATACTCGAGTATGCGACCGTAAAGAGCTACGGTGTTTCGTCCTTTCC |
| shSOSTDC1-F4 | ATCTCGAGATAAAGGATTTCTGTGGCATCTTTTTTGAATTCTCGACCTCGAG |
| shSOSTDC1-R4 | CTTTATCTCGAGATAAAGGATTTCTGTGGCATCGGTGTTTCGTCCTTTCC |
| shCHD1-F1 | AACTCGAGTTATAGCTCTTGATAAACCGCTTTTTGAATTCTCGACCTCGAG |
| shCHD1-R1 | CTATAACTCGAGTTATAGCTCTTGATAAACCGCGGTGTTTCGTCCTTTCC |
| shCHD1-F2 | AACTCGAGTTTGCCAGGAAGTAAGAGTGGTTTTTGAATTCTCGACCTCGAG |
| shCHD1-R2 | GGCAAACTCGAGTTTGCCAGGAAGTAAGAGTGGCGGTGTTTCGTCCTTTCC |
| shKPNA1-F2 | ATCTCGAGATCTGATAGATATGAGAGGGCTTTTTGAATTCTCGACCTCGAG |
| shKPNA1-R2 | TCAGATCTCGAGATCTGATAGATATGAGAGGGCGGTGTTTCGTCCTTTCC |
| shKPNA1-F3 | ATCTCGAGATGCTCAATAAGATCAAAGGCTTTTTGAATTCTCGACCTCGAG |
| shKPNA1-R3 | GAGCATCTCGAGATGCTCAATAAGATCAAAGGCGGTGTTTCGTCCTTTCC |
| shKPNA2-F1 | TTCTCGAGAAAGATTAGAAAGTGTCCAGGTTTTTGAATTCTCGACCTCGAG |
| shKPNA2-R1 | ATCTTTCTCGAGAAAGATTAGAAAGTGTCCAGGCGGTGTTTCGTCCTTTCC |
| shKPNA2-F2 | TTCTCGAGAAATTTCGGAATCAAACCAGCTTTTTGAATTCTCGACCTCGAG |
| shKPNA2-R2 | AAATTTCTCGAGAAATTTCGGAATCAAACCAGCGGTGTTTCGTCCTTTCC |
| shKPNA3-F1 | TACTCGAGTAATCCAGCATCTATTACAGCTTTTTGAATTCTCGACCTCGAG |
| shKPNA3-R1 | GGATTACTCGAGTAATCCAGCATCTATTACAGCGGTGTTTCGTCCTTTCC |
| shKPNA3-F4 | AACTCGAGTTCACAAACATTCTGATGTGGTTTTTGAATTCTCGACCTCGAG |
| shKPNA3-R4 | TGTGAACTCGAGTTCACAAACATTCTGATGTGGCGGTGTTTCGTCCTTTCC |

F, forward primer; R, reverse primer

**Table S3. The sequences of primers used for qRT-PCR.**

| Gene | Forward (5'-3') | Reverse (5'-3') |
| --- | --- | --- |
|  |  |  |
| TBP | TGCACAGGAGCCAAGAGTGAA | CACATCACAGCTCCCCACCA |
| SOSTDC1 | AGCTCCTTCAGAGGGCTGAT | GGAGGCAGGCATTTCAGTAA |
| CHD1 | GCAGTGACTCTGACTCCGGATCT | AGTCAGAGTCACTGCTACCTGAC |
| BTRC | CCAGACTCTGCTTAAACCAAGAA | GGGCACAATCATACTGGAAGTG |
| KPNA1 | ATTCAAGCGGAGAAATGTTGCT | ACTCCTGGTGTGCTGATAACT |
| KPNA2 | ACATAATCCGGGCTGGTTTGA | GGGAGATGCCAACAGAGAAATG |
| KPNA3 | ACAGAAATCCACCGATTGATGAC | GGTACTGCATTAGACTGCACAAC |
